# Exercise-induced anti-inflammatory adaptation in older adults: a Bayesian network meta-analysis and dose–response analysis of C-reactive protein

**DOI:** 10.3389/fmed.2026.1889076

**Published:** 2026-08-06

**Authors:** Fuxin Hong, Long Wang, Zhen Wan, Daehee Kim, Jung Woon Seo

**Affiliations:** 1 Department of Physical Education, Pukyong National University, Busan, Republic of Korea; 2 Kyung Hee University Graduate School of Physical Education, Yongin, Republic of Korea

**Keywords:** C-reactive protein, dose-response relationship, inflammaging, interleukin-6, model-based network meta-analysis, network meta-analysis, older adults

## Abstract

**Background:**

Global population aging is increasing the number of older adults living with chronic diseases and functional limitations. Chronic low-grade inflammation is common in later life and is associated with multisystem decline and age-related disease risk. C-reactive protein (CRP) and interleukin-6 (IL-6) are widely used circulating biomarkers in this context. Exercise may help modulate inflammatory biomarkers, but existing evidence remains heterogeneous across exercise modalities, prescriptions, and outcomes.

**Methods:**

This study adhered to the PRISMA-NMA guidelines (PROSPERO: CRD420261397178). Six major databases (PubMed, Embase, Web of Science, Scopus, EBSCOhost, and Cochrane Library) were searched from inception to May 2026. Fifty-four randomized controlled trials (RCTs) enrolling 2,836 participants aged ≥60 years were included. A Bayesian random-effects network meta-analysis (NMA) was conducted using standardized mean differences (SMDs) to compare five exercise modalities: aerobic exercise (AE), resistance training (RT), combined training (CT), mind-body exercise (MBE), and multi-component exercise (MCE). A model-based network meta-analysis (MBNMA) was further constructed with weekly metabolic equivalent minutes (MET-min·week^−1^) as a continuous exposure variable and a quadratic function to model non-linear dose-response relationships. Reference coding was applied to isolate baseline drift from natural aging. Spearman rank correlation between CRP and IL-6 effect estimates was assessed strictly at the within-study level.

**Results:**

The evidence network demonstrated satisfactory overall consistency; network meta-regression did not identify baseline disease status as a statistically significant moderator of the dose-response curve (*P* > 0.05). NMA suggested modality-specific anti-inflammatory patterns: AE retained the most favorable ranking for CRP reduction (SUCRA = 0.89; SMD = −0.83, 95% CrI: −1.42 to −0.24). In the primary IL-6 network, MBE ranked highly; however, influence analysis identified Ali Ismail 2025 as an extreme-effect study. After excluding this study, the MBE 900 MET-min week^−1^ node lost its favorable ranking (SUCRA 0.845 to 0.415; probability of being best 0.291 to 0.035), and the estimated effect shifted toward the null (median −5.50 to 0.62). MBNMAdose suggested non-linear dose-response patterns, but Hellinger distances indicated substantial posterior divergence between common-effect and random-effects models. These findings indicate that IL-6 modality rankings, particularly those involving MBE, should be interpreted cautiously.

**Conclusions:**

Exercise interventions are associated with improvements in inflammatory biomarkers among older adults, with effects varying by modality and dose. AE showed the most consistent signal for CRP reduction. The apparent MBE advantage for IL-6 was not robust to exclusion of an influential study and should be treated as exploratory rather than definitive. Dose-response findings suggest a probable moderate-dose benefit region, but credible-interval uncertainty and substantial Bayesian heterogeneity indicate that dose regions should be interpreted cautiously rather than as fixed clinical thresholds.

## 1 Introduction

The accelerating pace of global population aging has increased the number of older adults living with chronic diseases and functional limitations (1). Chronic low-grade inflammation, often described as inflammaging, is commonly observed in later life and is associated with multisystem functional decline, cardiometabolic disease, sarcopenia, neuroinflammatory processes, and cognitive or mood-related outcomes (2–5). These associations do not imply that population aging itself directly causes inflammatory disease burden; rather, inflammation represents one biological pathway through which aging-related vulnerability may be linked to adverse health outcomes. Therefore, identifying feasible non-pharmacological strategies that may help modulate inflammatory biomarkers in older adults remains an important public-health question.

Exercise intervention, owing to its multisystem benefits, has been widely endorsed by the World Health Organization (WHO) (6). Conventional pairwise meta-analyses have established that regular exercise can reduce peripheral inflammation in older adults through mechanisms including improved adipose tissue distribution, decreased visceral fat accumulation, and downregulation of the NF-κB signaling pathway (7, 8). Nevertheless, existing systematic reviews present considerable heterogeneity, with some studies reporting statistically non-significant or mixed effects (9). The primary source of this evidentiary conflict is the highly complex structural heterogeneity of exercise interventions: anti-inflammatory effects are not only discretely modulated by exercise modality (aerobic, resistance, combined, mind-body, and multi-component) but are also non-linearly constrained by the “continuous dose” space defined by the interaction of frequency, intensity, and session duration. Yet most existing multi-exercise comparisons have remained at the level of discrete categorical classification, without systematic modeling within a unified continuous dose space.

Exercise dose is fundamentally a continuous variable. According to the Compendium of Physical Activities, metabolic equivalents (METs) for different exercise modalities—particularly those incorporating resistance training and respiratory regulation—exhibit substantial environmental dependence and individual heterogeneity in older populations, rendering conventional categorical grouping susceptible to systematic measurement error (10). Furthermore, the relationship between exercise dose and immune response is not simply linear; evidence suggests the existence of an “efficacy threshold” and “plateau effect” attributable to insufficient stimulus or excessive physiological stress (11). In the complex real-world context of older adults—encompassing healthy individuals, those with depression, and patients with multiple chronic comorbidities—the optimal anti-inflammatory dose boundary of exercise remains without consensus (12).

From a biological perspective, exercise-induced IL-6 can act as a transient myokine involved in metabolic regulation and anti-inflammatory signaling, whereas CRP reflects downstream hepatic acute-phase activity over longer time windows (13, 14). Therefore, CRP and IL-6 may not change synchronously across all exercise modalities, intensities, intervention durations, and follow-up windows. In this manuscript, biomarker decoupling is used descriptively to denote partial discordance between CRP and IL-6 response patterns; it is not used as evidence of a confirmed mechanistic separation. Three core evidence gaps currently exist: (1) absence of unified continuous-dose modeling across multiple exercise modalities within an inclusive older adult population framework; (2) limited ability of conventional NMA to characterize non-linear dose-response patterns; and (3) limited probabilistic evidence on whether CRP and IL-6 responses align at the study level.

In response to these gaps, this study constructs a dual-track integrative framework combining Bayesian NMA and MBNMA based on RCT evidence, with the following aims: (1) compare the relative efficacy of five major exercise modalities in improving CRP and IL-6 in older adults; (2) characterize the non-linear structure of continuous dose-response curves; (3) estimate dose regions associated with potentially greater anti-inflammatory benefit while explicitly accounting for uncertainty; and (4) explore the association between CRP and IL-6 responses at the within-study level. Our core hypotheses are that: (1) the five exercise modalities may exhibit outcome-specific anti-inflammatory patterns; (2) exercise dose and inflammatory improvement may exhibit a non-linear relationship; and (3) CRP and IL-6 responses may be only partially aligned across studies, reflecting potentially distinct regulatory pathways in exercise-induced anti-inflammatory adaptation among older adults.

## 2 Methods

### 2.1 Study protocol and registration

**Figure 1:**
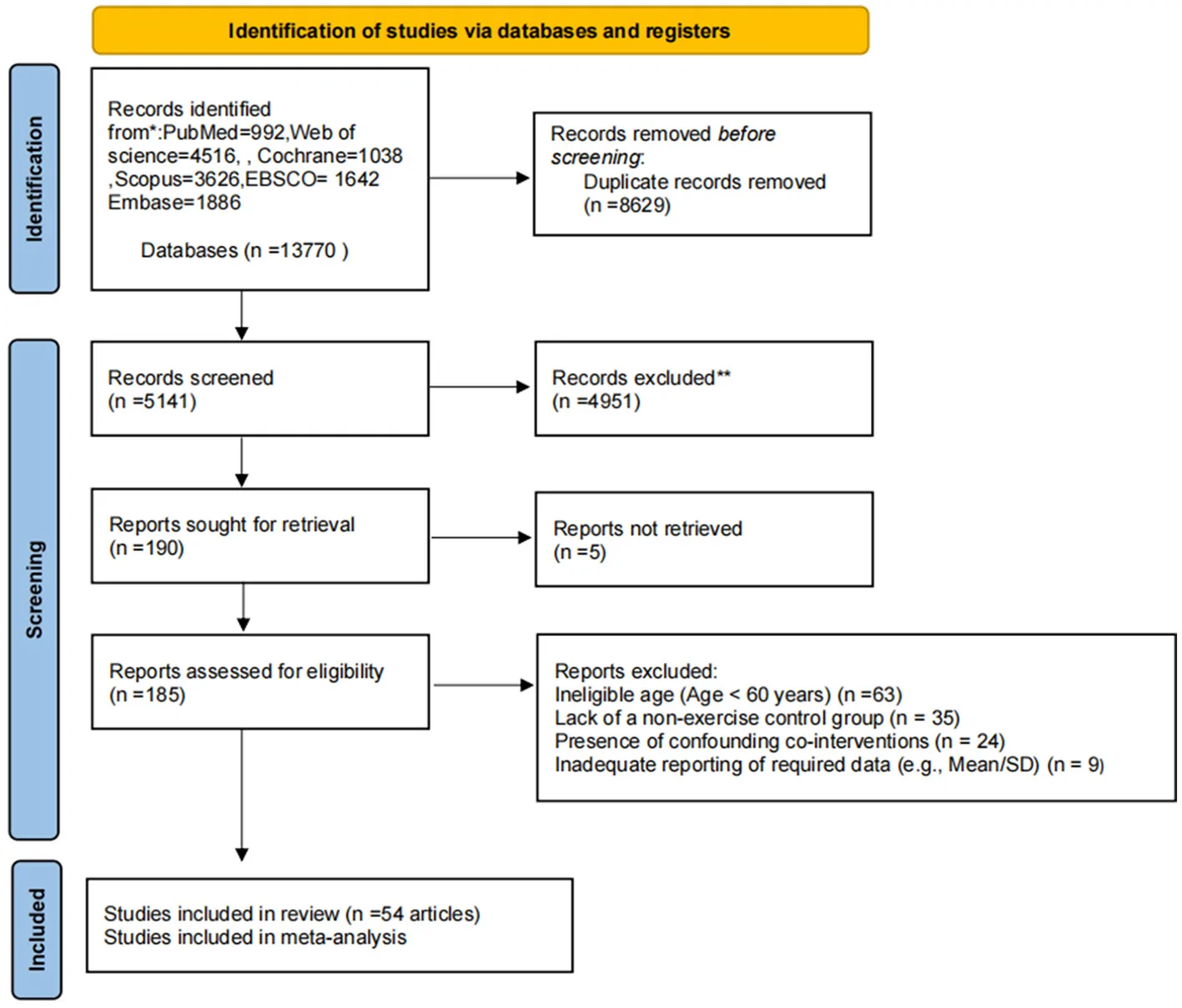
PRISMA flowchart of included studies.

This systematic review and network meta-analysis was conducted in accordance with the Preferred Reporting Items for Systematic Reviews and Meta-Analyses extension for Network Meta-Analyses (PRISMA-NMA) (15) and the Cochrane Handbook for Systematic Reviews of Interventions (16). The study protocol was prospectively registered in the PROSPERO database (registration number: CRD420261397178). As this study involved secondary analysis of publicly available published data, neither ethical approval nor informed consent was required. The literature screening process was reported according to the PRISMA flow diagram (Figure 1).

### 2.2 Search strategy

PubMed, Embase, Web of Science, Scopus, EBSCOhost, and the Cochrane Library were systematically searched from database inception to May 2026. The search strategy combined Medical Subject Headings (MeSH) terms with free-text keywords, including: “exercise,” “physical activity,” “aerobic exercise,” “resistance training,” “C-reactive protein,” “CRP,” “interleukin-6,” and “IL-6,” connected using Boolean operators (AND/OR). Complete search strategies for each database are provided in Supplementary File 1. In addition, reference lists of all included studies and relevant systematic reviews were manually screened to minimize the risk of missing eligible studies. No restrictions were applied with regard to language, geographic region, or publication status.

Search strategies were independently developed by two investigators (WL and FX-H) and reviewed by a senior researcher (WZ) with systematic review expertise to ensure comprehensiveness and reproducibility. The same two investigators independently screened titles and abstracts, followed by full-text eligibility assessment. Discrepancies were resolved by a third investigator through discussion and consensus. All references were managed using EndNote X9. Inter-rater agreement during the screening process was assessed using Cohen's kappa coefficient. Kappa was calculated for the full-text eligibility assessment among articles for which full texts were successfully retrieved. Articles that could not be retrieved were recorded separately and were not included in the kappa calculation.

### 2.3 Eligibility criteria

#### 2.3.1 Inclusion criteria (PICOS framework)

**(P) Population:** Older adults with a mean age ≥60 years, regardless of sex or health status.

**(I) Intervention:** Any planned exercise intervention, including comparisons between a single exercise modality and a control condition, or head-to-head comparisons between different modalities. Exercise was defined as planned, purposeful, and repeatable physical activity (17), and categorized into five types:

(1) Aerobic exercise (AE): exercise primarily targeting cardiorespiratory endurance and aerobic metabolic capacity, including walking, jogging, cycling, swimming, and dance-based continuous activities;

(2) Resistance training (RT): exercise primarily targeting muscular strength, muscular endurance, and skeletal muscle function, including machine-based training, elastic band training, dumbbell training, and bodyweight resistance exercises;

(3) Combined training (CT): interventions combining two distinct exercise modalities simultaneously, most commonly aerobic exercise plus resistance training, to improve both cardiorespiratory function and muscular function concurrently;

(4) Mind-body exercise (MBE): exercise emphasizing the integration of physical movement, breathing regulation, and mental focus, including Tai Chi, yoga, Ba Duan Jin (Eight Brocades), and Qigong;

(5) Multi-component exercise (MCE): comprehensive training incorporating three or more exercise components, typically including aerobic training, resistance training, balance training, and flexibility training, to broadly improve physical function and health outcomes in older adults.

**(C) Comparator:** Usual care, health education, maintenance of habitual lifestyle, or no exercise intervention.

**(O) Outcomes:** At least one reported measure of change in CRP (including high-sensitivity CRP [hs-CRP]) and/or IL-6.

**(S) Study design:** Randomized controlled trials (RCTs) with an intervention duration ≥4 weeks.

Exclusion criteria: Acute exercise studies (duration < 4 weeks), non-randomized controlled studies, crossover designs, conference abstracts, study protocols, review articles, and studies from which effect sizes could not be extracted.

For multi-arm trials in Bayesian NMA and MBNMA, original multi-arm data were incorporated directly, with between-arm correlations automatically adjusted by the model based on a multivariate normal distribution.

### 2.4 Data extraction

Two investigators (WL and FX-H) independently extracted the following information: first author and publication year, sample size, age and sex distribution, health status, exercise modality, intervention duration, exercise frequency, session duration, exercise intensity, and baseline and endpoint measurements (mean and SD) for CRP and IL-6. Where primary data were incomplete, the corresponding authors were contacted by email to request supplementary information.

### 2.5 Exercise dose coding

Exercise dose was quantified uniformly in MET-min·week^−1^ using the formula: Dose = METs × Duration (min) × Frequency (sessions/week). MET values were assigned based on exercise-type-specific coding standards from the 2011 Compendium of Physical Activities (10).

For studies that did not explicitly report session duration, the mean value from studies of the same exercise type was used for imputation. For progressive training protocols, the average exercise dose across the entire intervention period was applied. In subsequent MBNMA analyses, all exercise doses were entered as continuous variables without artificial categorization or discretization.

### 2.6 Risk of bias and evidence quality assessment

Three investigators independently assessed the methodological quality of included studies using the Cochrane Risk of Bias tool version 1 (RoB 1) (18), evaluating the following domains: random sequence generation, allocation concealment, blinding of participants and personnel, blinding of outcome assessors, incomplete outcome data, selective outcome reporting, and other sources of bias. Each domain was rated as “low risk,” “high risk,” or “unclear risk.” Disagreements were resolved through discussion or adjudication by a fourth investigator. Results were visualized using Review Manager 5.4. Given the inherent nature of exercise interventions, blinding of participants and personnel was rated as high risk in the majority of studies.

Evidence quality for NMA results was assessed using the GRADE (Grading of Recommendations Assessment, Development and Evaluation) framework (19), considering study limitations, indirectness, transitivity, inconsistency, imprecision, and publication bias. Evidence quality was rated as high, moderate, low, or very low.

### 2.7 Outcome measurement

Given the heterogeneity of clinical measurement units for CRP (including hs-CRP) and IL-6 across studies (e.g., mg/L, mg/dL, pg/mL), mean difference (MD) was adopted as the unified effect measure for both NMA and MBNMA, preserving absolute clinical changes in inflammatory markers and enhancing the interpretability of dose-response curve estimates.

All measurement units were standardized to metric equivalents during data extraction: CRP was converted to mg/L and IL-6 to pg/mL. For skewed data reported as median and interquartile range (IQR), the Wan and McGrath formulas were applied to estimate means and standard deviations (SDs).

For studies with missing change-score standard deviations (SD_change), variance was imputed using the Cochrane Handbook recommended formula, with a baseline-to-endpoint correlation coefficient (r) of 0.5 in the primary analysis. Sensitivity analyses were conducted with r set to 0.3 and 0.7 to evaluate the robustness of this assumption.

### 2.8 Statistical analysis

Bayesian NMA and MBNMA were prespecified as the primary inferential framework. This framework was selected because the objective of this review was not limited to estimating pooled effects within direct comparisons. Rather, the study aimed to compare multiple exercise modalities simultaneously, synthesize direct and indirect evidence within a coherent network, account for correlations arising from multi-arm trials, estimate posterior ranking probabilities, and extend the network framework to model continuous exercise dose-response relationships. Although frequentist synthesis of direct comparisons is statistically feasible given the number of included RCTs, it does not fully address the comparative ranking and dose-response objectives of this review within a single probabilistic hierarchical model.

#### 2.8.1 Network meta-analysis (NMA)

Following verification of network transitivity—assessed by evaluating the distributional balance of key covariates (age, sex ratio, baseline inflammatory levels, and intervention duration) across comparisons—a Bayesian random-effects NMA was performed. A network plot was constructed to visualize direct and indirect comparisons among exercise modalities.

The model was implemented using Markov Chain Monte Carlo (MCMC) methods with 4 parallel chains, each running 50,000–100,000 iterations; the first 20,000 iterations were discarded as burn-in, and a thinning interval of 10 was applied to reduce autocorrelation and improve posterior sampling stability. Model convergence was evaluated using the Brooks–Gelman–Rubin diagnostic, with a potential scale reduction factor (PSRF) approaching 1.0 indicating satisfactory convergence. A weakly informative prior was specified for the between-study heterogeneity parameter: τ ~ Uniform(0, 2). Global consistency was assessed by comparing the deviance information criterion (DIC) and residual deviance between consistency and inconsistency (UME) models; local inconsistency was evaluated using the node-splitting method.

#### 2.8.2 Model-based dose-response network meta-analysis (MBNMA)

Subject to satisfactory network connectivity, a Bayesian random-effects MBNMA was constructed to characterize the non-linear relationship between exercise dose and inflammatory adaptation. Candidate dose-response functions included: linear, Emax, first- and second-order fractional polynomial (FP1/FP2), and quadratic models.

Reference coding was applied in model construction, allowing the control group (zero dose) to estimate a relative intercept, thereby capturing and isolating the systematic influence of natural aging or habitual physical activity drift on baseline inflammation during the follow-up period, and producing more accurate dose-response estimates.

Model fit was comprehensively evaluated using DIC, residual deviance, and between-study heterogeneity parameters. The model with the minimum DIC and residual deviance closest to the network degrees of freedom was selected as the final interpretive framework. Effect sizes are expressed as posterior medians with 95% credible intervals (95% CrI), and 95% prediction intervals (95% PI) are reported to reflect the range of true effects under future heterogeneous conditions.

To quantify heterogeneity within the primary Bayesian MBNMAdose framework, Hellinger distance (D) was calculated between posterior beta.1 and beta.2 distributions from common-effect and random-effects quadratic MBNMAdose models. D ranges from 0 to 1, with larger values indicating greater posterior distributional divergence after allowing for between-study heterogeneity. The Hellinger diagnostic models used three chains, 20,000 iterations, 10,000 burn-in iterations, and thinning of 10, and convergence was evaluated using trace plots, autocorrelation plots, potential scale reduction factors (Rhat), and effective sample sizes. Duplicate arms with the same study, agent, and dose were combined using inverse-variance weighting; studies that became single-arm after this processing were excluded from the corresponding network analysis because they did not contribute comparative evidence.

#### 2.8.3 Clinical heterogeneity control and biomarker co-analysis

Because the IL-6 network contained one potentially influential MBE study (Ali Ismail 2025), an additional *post hoc* influence analysis was conducted in response to peer review. This analysis excluded Ali Ismail 2025 and refitted the IL-6 modality-level Bayesian NMA and quadratic MBNMAdose models. Changes in posterior effects, SUCRA, probability of being best, and dose-response parameters were compared with the full-data analysis.

To address the potential impact of participant health status heterogeneity on network transitivity, the node-splitting method was applied to quantify direct vs. indirect evidence differences across closed loops. Baseline health status (healthy older adults vs. patients with chronic disease) was incorporated as a key covariate in a Bayesian meta-regression to quantify its moderating effect on the exercise dose-response curve.

To explore macroscopic cooperative patterns of CRP and IL-6 improvement, bivariate SUCRA scatter plots were generated to visualize the relative efficacy rankings of the five exercise modalities across both outcomes. At the microscopic level, Spearman rank correlation analysis was conducted strictly within the 17 RCTs that simultaneously reported both outcomes, using observed change values extracted from each intervention arm to assess whether CRP and IL-6 responses were aligned at the study level.

## 3 Results

### 3.1 Literature screening

Initial database searches identified 13,770 records. After duplicate removal, 5,141 records entered title and abstract screening, of which 4,951 were excluded for clearly not meeting the inclusion criteria. Full texts were sought for 190 potentially eligible reports. Five full-text reports could not be retrieved despite retrieval attempts; therefore, 185 full-text articles were assessed for eligibility by two independent reviewers. Inter-rater agreement before consensus discussion was high, with an observed agreement of 0.946, an expected agreement of 0.582, and a Cohen's kappa of 0.871, indicating almost perfect agreement. The contingency table and calculation details are provided in Supplementary Table S3. After consensus discussion, 54 RCTs involving 2,836 older participants were ultimately included in the quantitative synthesis (20–73). The PRISMA flow diagram is presented in Figure 1.

**Figure 2:**
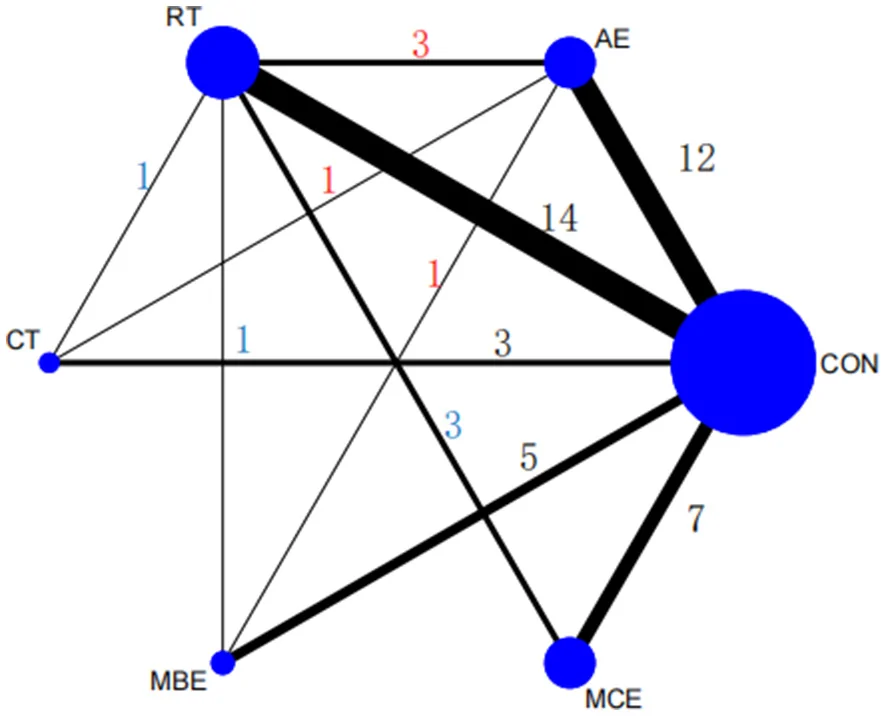
Network plot of comparisons for all studies included in the network meta-analysis. Line width is proportional to the number of direct comparison effect estimates and node size is proportional to the number of participants.

Among included studies, 39 reported CRP or hs-CRP outcomes, 32 reported IL-6 outcomes, and 17 reported both. The overall evidence network demonstrated good connectivity; however, direct comparisons between exercise modalities were unevenly distributed—AE and RT were supported by more studies, while direct evidence for MBE and MCE was comparatively limited. The network structure is depicted in Figure 2.

### 3.2 Characteristics of included studies

The 54 included RCTs enrolled 2,836 older participants with a mean age of 76.98 years. Exercise frequency ranged from 1 to 5 sessions per week, with intervention durations spanning 6 weeks to 12 months; approximately 18.5% of studies had a 12-week intervention period. Participants were predominantly older adults with chronic disease (41 studies), with the remainder comprising healthy older adults (7 studies) and populations with mixed or unspecified health status (6 studies).

Exercise modalities included AE, RT, CT, MBE, and MCE, with AE and RT being the most prevalent; MBE and MCE were represented by fewer studies. Exercise doses ranged from approximately 120 to 1,800 MET-min·week^−1^, with most studies concentrated in the moderate dose range (approximately 450–900 MET-min·week^−1^).

With respect to inflammatory outcome measurement, 25 studies used CRP and 14 used hs-CRP; IL-6 was predominantly measured as plasma or serum concentration. Complete characteristics of included studies and interventions are provided in Supplementary Tables S1, S6.

### 3.3 Risk of bias and evidence quality

All 54 included studies (100%) were rated as low risk of bias for random sequence generation. Twenty studies were rated as low risk for allocation concealment; the remainder were rated as “unclear risk” due to insufficient reporting. Given the practical difficulties of blinding in exercise intervention research, the vast majority of studies were rated as high risk for blinding of participants and personnel. Eighteen studies were rated as low risk for blinding of outcome assessors. Three studies were rated as high risk for incomplete outcome data due to substantial attrition (21%−43%). Risk of bias assessment results are provided in Supplementary File S3.

GRADE assessment indicated that evidence quality was predominantly low to very low for most comparisons, primarily attributable to high between-study heterogeneity, limited sample sizes for certain comparisons, wide credible intervals, and insufficient direct evidence for some modalities. Detailed results are provided in Supplementary File S4.4.

### 3.4 Network meta-analysis—CRP outcomes

Network structure analysis identified AE-vs.-control as the primary source of direct comparison evidence; direct comparisons involving MBE and MCE were relatively scarce, though overall network connectivity was sufficient to support Bayesian NMA.

SUCRA rankings indicated that AE ranked highest for CRP improvement (SUCRA = 0.89), followed by RT (0.68), CT (0.60), MBE (0.46), and MCE (0.16). In terms of effect sizes, AE demonstrated a consistent CRP-lowering trend (SMD = −0.73, 95% CrI: −1.17 to −0.29); RT also showed meaningful improvement (SMD = −0.46, 95% CrI: −0.84 to −0.08). Credible intervals for CT (SMD = −0.42, 95% CrI: −1.26 to 0.42) and MBE (SMD = −0.24, 95% CrI: −0.91 to 0.42) crossed zero, and MCE showed no clear improvement trend (SMD = 0.09, 95% CrI: −0.43 to 0.61).

Model fit was satisfactory for the random-effects consistency model (DIC = 128.1). The DIC difference between the consistency and UME models was < 5, indicating good overall network consistency. Node-splitting analyses revealed no significant local inconsistency (all P > 0.05). Detailed results are provided in Supplementary Files S4.2, S4.5.

### 3.5 Dose-response analysis—CRP

**Figure 3:**
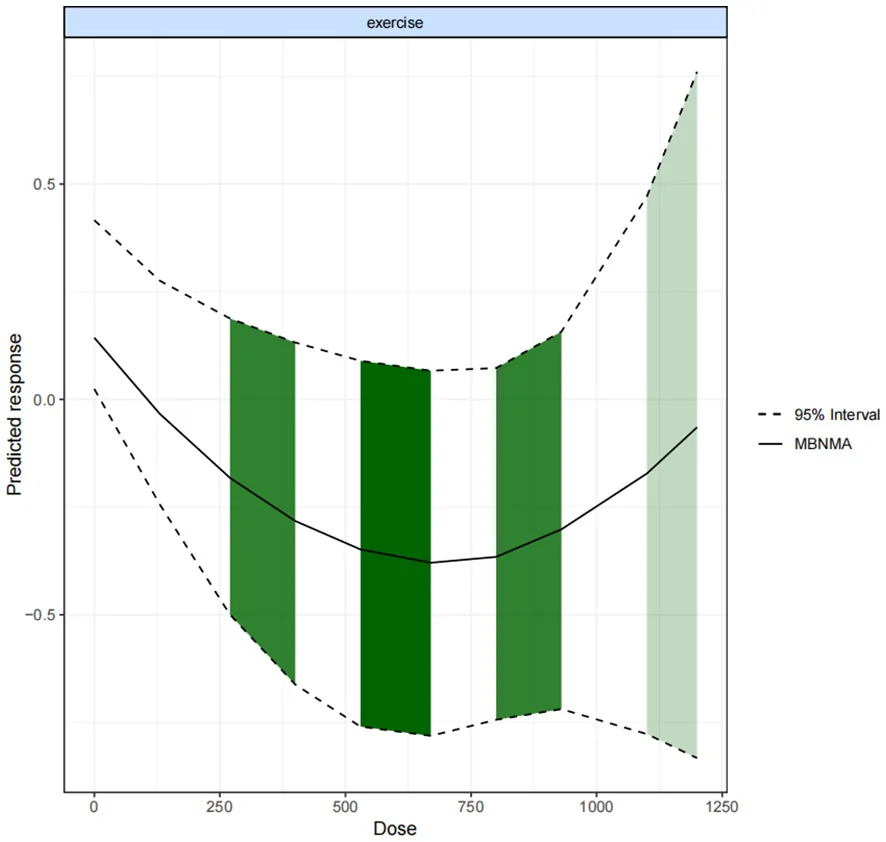
Dose-response relationship between overall physical activity levels and changes in CRP among older adults.

Bayesian MBNMA was applied to explore the dose-response relationship between exercise and CRP change. Linear, Emax, restricted cubic spline (RCS), and quadratic models were constructed and compared by DIC. The quadratic model provided the best fit (DIC = 105.0), outperforming the linear model (DIC = 107.9), indicating a non-linear dose-response relationship between exercise and CRP (Figure 3).

The overall dose-response curve showed a general downward trend in CRP with increasing exercise dose, with a potentially more favorable trend in the moderate-to-moderately-high dose range. The largest estimated effect occurred at approximately 690 MET-min·week^−1^ (MD = −0.370, 95% CrI:−0.779 to 0.063); beyond 1,200 MET-min·week^−1^, the curve appeared to plateau. The 95% CrI crossed zero at all dose levels, indicating residual uncertainty in current evidence.

Modality-stratified analysis revealed: AE showed a potentially favorable improvement trend in the 300–600 MET-min·week^−1^ range, with the largest estimated effect at 560 MET-min·week^−1^ (MD = −0.860, 95% CrI: −1.507 to −0.201); RT showed its largest estimated effect in the 600–900 MET-min·week^−1^ range, with the largest estimate at 900 MET-min·week^−1^ (MD = −0.617, 95% CrI: −1.453 to 0.224); CT showed a mild overall downward trend without clear additional improvement at high doses; MBE and MCE exhibited unstable dose-response patterns without clear dose-dependent changes. Overall, moderate-dose exercise may represent a potentially favorable CRP benefit region compared with higher doses, although most credible intervals crossing zero warrant cautious interpretation.

### 3.6 IL-6 analysis

#### 3.6.1 Network meta-analysis—IL-6 outcomes

In the primary IL-6 NMA, MBE initially showed the most favorable ranking (SUCRA = 0.87; SMD = −1.18, 95% CrI: −2.24 to −0.12). However, the model-ready dataset showed that this ranking was strongly influenced by Ali Ismail 2025. In that study, the extracted MBE change value was −14.08 and the control change value was 1.20, producing a direct MBE-control contrast of −15.28 (SE = 2.15, z = −7.10). After excluding this study, the MBE 900 MET-min week^−1^ node changed from median −5.50 (95% CrI −12.03 to 0.94), SUCRA 0.845, and probability of being best 0.291 to median 0.62 (95% CrI −6.67 to 7.74), SUCRA 0.415, and probability of being best 0.035. Therefore, MBE should no longer be interpreted as robustly superior for IL-6 reduction.

Consistency testing indicated a DIC difference of < 5 between consistency and UME models (133.0 vs. 135.0), supporting good overall network consistency. Node-splitting analyses revealed no significant local inconsistency (all *P* > 0.05).

#### 3.6.2 2 Dose-response analysis—IL-6

Overall dose-response analysis (Supplementary File S5.2) indicated a general downward trend in IL-6 with increasing exercise dose, with moderately high doses showing a potentially more favorable improvement probability. The largest estimated effect occurred at approximately 1,500 MET-min·week^−1^ (MD = −1.116, 95% CrI: −2.574 to 0.357), though most credible intervals crossed zero.

Modality-stratified dose-response analysis initially suggested a favorable MBE-related IL-6 pattern in moderate dose ranges; however, this pattern was not robust after excluding Ali Ismail 2025. For MBE beta.1 (5), the posterior median changed from −0.0141 (95% CrI −0.0391 to 0.0108; posterior probability that beta.1 (5) < 0 = 0.877) to 0.0006 (95% CrI −0.0224 to 0.0240; posterior probability that beta.1 (5) < 0 = 0.479). For MBE beta.2 (5), directional support similarly attenuated. These findings indicate that the apparent MBE dose-response signal was sensitive to the outlying study rather than a stable network-wide pattern.

### 3.7 Bayesian heterogeneity assessment and MCMC diagnostics

The quadratic MBNMAdose-based heterogeneity assessment showed marked posterior divergence between common-effect and random-effects models. Median Hellinger D values were 0.645 for the CRP modality-level model, 0.816 for the CRP overall exercise dose-node model, 0.809 for the IL-6 modality-level model, and 0.811 for the IL-6 overall exercise dose-node model. The corresponding between-study SD estimates were 2.319 (95% CrI 1.759 to 3.153), 2.309 (95% CrI 1.775 to 3.040), 4.101 (95% CrI 3.103 to 5.637), and 3.812 (95% CrI 2.917 to 5.082), respectively. Random-effects models had substantially lower DIC values than common-effect models across all analysis sets. Trace and autocorrelation plots did not indicate problematic chain behavior; coda-based Rhat values were < = 1.002, with minimum effective sample sizes above 2,790.

### 3.8 Influence analysis excluding Ali Ismail 2025

The data audit confirmed that the IL-6 value for Ali Ismail 2025 was extracted as a change-from-baseline value rather than an endpoint value: the MBE arm changed from 19.12 to 5.04, corresponding to −14.08 in the model-ready dataset. No log transformation was applied, and IL-6 units were retained as pg/mL after standardization. Although this value was consistent with the extracted table, it was statistically influential. Excluding this study reduced the MBE 900 MET-min week^−1^ SUCRA from 0.845 to 0.415 and the probability of being best from 0.291 to 0.035. In the MBNMAdose model, the MBE linear dose parameter beta.1 (5) shifted from −0.0141 to 0.0006, indicating loss of directional support. Full influence-analysis tables and trace/autocorrelation plots are provided in Supplementary File S9.

### 3.9 Exploratory co-analysis of CRP and IL-6

To investigate the potential association between different inflammatory biomarkers during exercise-induced anti-inflammatory adaptation, an exploratory co-analysis of CRP and IL-6 was conducted.

Macroscopic level (network-wide ranking): Bivariate SUCRA scatter plots (Supplementary File S6) suggested outcome-specific ranking patterns, with AE performing best for CRP and CT showing a relatively favorable combined position. The IL-6 component of the ranking, however, was sensitive to Ali Ismail 2025 and should be interpreted as unstable. Spearman rank correlation between SUCRA rankings for the two outcomes was moderate (Spearman rho = 0.43, *P* = 0.42), not reaching statistical significance, indicating partial but incomplete synchrony in network-wide ranking patterns.

**Figure 4:**
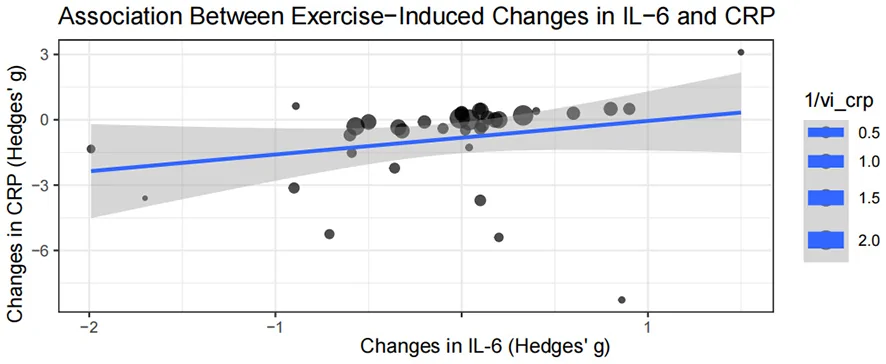
Robust meta-regression plot showing the association between exercise-induced alterations in Hedges' g for IL-6 and CRP.

Microscopic level (within-study analysis): Among the 17 RCTs simultaneously reporting both outcomes, exercise-induced changes in IL-6 and CRP generally co-varied in a positive direction (Figure 4). Most study data points clustered near the null effect, while a minority of studies exhibited large IL-6 effects that widened the distal credible intervals and affected model stability. Therefore, the co-analysis should be regarded as exploratory evidence of possible biomarker co-response rather than proof of a stable cooperative relationship.

In the exploratory dose-response analysis with exercise dose as a continuous variable (Supplementary File S6), no clear linear relationship was observed between CRP improvement and increasing exercise dose; the overall regression trend approached horizontal, suggesting that CRP changes may be more substantially influenced by exercise modality, baseline inflammatory status, and participant health background than by exercise dose *per se*.

Overall, exercise intervention may simultaneously modulate both CRP and IL-6 through shared inflammatory regulatory pathways, though the association between the two biomarkers is limited, and definitive linear dose-effect evidence is lacking. Given the constraints of study numbers and the influence of extreme values, these findings require validation in future high-quality research.

## 4 Discussion

Using Bayesian NMA and MBNMA, this study systematically evaluated the effects of different exercise modalities and continuous exercise doses on CRP and IL-6 in older adults, and further explored the potential association between these two inflammatory biomarkers. Results suggested modality-specific anti-inflammatory patterns: AE ranked highest for CRP reduction, whereas the apparent MBE advantage for IL-6 was not robust after exclusion of Ali Ismail 2025. Dose-response analysis indicated a non-linear pattern in which anti-inflammatory benefits did not appear to increase indefinitely with dose. However, the MBNMAdose-based Hellinger distance analysis indicated substantial posterior divergence between common-effect and random-effects models, supporting cautious interpretation of dose windows and treatment rankings under between-study heterogeneity.

The highest SUCRA ranking of AE for CRP improvement, observed in the Bayesian NMA, aligns with the existing literature. Aerobic exercise may reduce chronic low-grade inflammatory burden through improvements in adipose metabolism, reduction of visceral fat accumulation, and enhanced insulin sensitivity (18, 74). Regular long-term aerobic exercise may also suppress NF-kB pathway activation and monocyte pro-inflammatory phenotype expression, thereby reducing production of CRP and other acute-phase proteins (19). The systematic review by Kasapis and Thompson similarly reported that moderate-intensity regular aerobic exercise was associated with lower circulating CRP levels (75). In the present study, AE showed a favorable CRP ranking across the network, but this ranking should be interpreted alongside credible-interval uncertainty and the substantial heterogeneity indicated by Hellinger D.

The initial high ranking of MBE for IL-6 should be interpreted as hypothesis-generating rather than conclusive. Exercise-induced IL-6 is not equivalent to the pro-inflammatory IL-6 observed in pathological states; IL-6 released by skeletal muscle during exercise may function as a myokine involved in metabolic regulation and anti-inflammatory signaling (13, 14). Breathing regulation, mental focus, and sustained low-intensity movement in MBE may plausibly influence autonomic activity, perceived stress, or hypothalamic-pituitary-adrenal (HPA) axis regulation (76–78). Nevertheless, the present network meta-analysis cannot directly test these mechanisms, and the exclusion analysis showed that the MBE ranking for IL-6 was sensitive to Ali Ismail 2025. Accordingly, these biological pathways are presented only as plausible explanations requiring direct mechanistic validation.

CT demonstrated relatively stable combined improvement effects across both CRP and IL-6. Whereas aerobic exercise primarily exerts anti-inflammatory effects through improvements in cardiorespiratory metabolic function, resistance training more specifically targets the maintenance of skeletal muscle mass and improvement of muscle metabolic status (79). CT integrates both exercise stimuli and may therefore yield synergistic benefits in improving muscle mass, mitochondrial function, and metabolic homeostasis (80). Libardi et al. (81) found that combined training more effectively reduced TNF-α and IL-6 compared to single exercise modalities, suggesting that CT may be more appropriate than any single modality as a comprehensive exercise strategy for long-term inflammatory management in older adults.

Regarding the dose-response relationship, the MBNMA results support a probable non-linear pattern: CRP and IL-6 tended to decline with increasing exercise dose, but additional benefit appeared to attenuate at higher doses. Model estimates suggest that anti-inflammatory benefit may emerge around the moderate-dose range and may plateau beyond approximately 1,200–1,300 MET-min·week^−1^. Because several dose-specific credible intervals crossed zero and Hellinger D values indicated substantial heterogeneity, these thresholds should be interpreted as hypothesis-generating dose regions rather than definitive clinical cut-points. This pattern is broadly consistent with the “J-curve” or inverted-U hypothesis of exercise immunology, whereby moderate exercise may confer anti-inflammatory protection while excessively high doses may be accompanied by transient immune stress or recovery burden (11, 82).

Notably, the 95% CrI widened substantially for predicted effects at doses exceeding 1,200–1,300 MET-min·week^−1^, reflecting both statistical and clinical considerations. Statistically, the relative scarcity of RCT evidence at extreme high doses increases model extrapolation variance in the distal region of the curve. Clinically, considerable individual variation in tolerance to high-dose exercise among older populations further compounds between-study heterogeneity. Campbell and Turner noted that excessive exercise may provoke transient immune stress and inflammatory fluctuation, potentially undermining long-term anti-inflammatory adaptation (83), underscoring the need for cautious interpretation of potential anti-inflammatory effects at very high exercise doses.

The exploratory co-analysis of CRP and IL-6 indicated a moderate positive correlation between SUCRA rankings that did not reach statistical significance. This finding suggests possible partial commonality in network-wide improvement patterns, but it does not establish synchronized or decoupled biomarker regulation. A cautious interpretation is that exercise effects on CRP and IL-6 may partly differ across modalities and biological pathways, consistent with the distinction between acute myokine signaling and longer-term peripheral inflammatory adaptation (79, 80). Consequently, changes in a single inflammatory marker should not be assumed to fully predict improvement in the other. Future studies incorporating larger-scale primary data, bivariate NMA frameworks, or individual participant data meta-analysis will be needed to clarify the joint probability distribution of inflammatory biomarker responses.

### 4.1 Strengths

This study integrates both NMA and MBNMA, enabling simultaneous comparison of exercise modality-specific effects and quantification of continuous dose-response relationships, thereby enhancing the clinical interpretive value of findings. To our knowledge, this is the first study to systematically investigate paired CRP and IL-6 response patterns within an older adult exercise-intervention evidence network. The Bayesian random-effects framework, supplemented by node-splitting, consistency testing, Hellinger distance heterogeneity assessment, and MCMC diagnostic plots, improves transparency regarding both model fit and uncertainty.

### 4.2 Limitations

RCT evidence at high exercise doses remains comparatively sparse, and the model 95% CrI widens substantially in extreme dose regions, partially crossing zero. Included studies exhibited clinical heterogeneity in participant health status, baseline inflammatory levels, intervention duration, and exercise intensity assessment methods; residual heterogeneity may affect result stability. The IL-6 MBE ranking was materially influenced by Ali Ismail 2025; after excluding this study, the MBE 900 MET-min week^−1^ node lost its favorable ranking and the dose-response signal shifted toward the null. Incomplete reporting of exercise intensity and session duration in some studies introduces potential error into dose estimates. The co-analysis of CRP and IL-6 is exploratory and conducted at the study level, subject to limitations imposed by study number and outlier influence; future high-quality research, ideally bivariate NMA or individual participant data meta-analysis, is warranted to more precisely delineate the joint regulatory mechanisms of exercise on the multidimensional inflammatory network in older adults.

## 5 Conclusions

Exercise interventions are associated with improvements in inflammatory biomarkers among older adults, with effects varying by exercise modality and dose. AE showed the most consistent favorable ranking for CRP reduction. For IL-6, the apparent MBE advantage in the primary analysis was not robust to exclusion of Ali Ismail 2025; therefore, no single exercise modality can be concluded as definitively best for IL-6 reduction based on the current evidence. Dose-response findings suggest a probable moderate-dose benefit region and attenuation of marginal benefit at higher doses; however, credible-interval uncertainty, substantial Bayesian heterogeneity, and sensitivity to influential studies indicate that these dose regions and treatment rankings should be interpreted cautiously. Individualized anti-inflammatory exercise prescriptions for older adults should therefore consider modality, dose, baseline health status, tolerance, and the current uncertainty of biomarker-specific evidence.
