# Supplementary material for: Exercise-induced anti-inflammatory adaptation in older adults: a Bayesian network meta-analysis and dose–response analysis of C-reactive protein
Source: Front Med (Lausanne). 2026 Aug 6;13:1889076. doi: 10.3389/fmed.2026.1889076 (PMC13489912; doi:10.3389/fmed.2026.1889076)
Supplement: Supplementary file 1 [file Data_Sheet_1.docx]

**Supplementary Materials**

Exercise-Induced Anti-Inflammatory Adaptation in Older Adults

Bayesian NMA, MBNMAdose, Hellinger Distance, and Reproducibility Materials

Template organization:

1. Search and screening reliability

2. Included-study characteristics

3. Risk of bias

4. Bayesian network meta-analysis, GRADE, and sensitivity analyses

5. Model-based dose-response network meta-analysis

6. Exploratory CRP and IL-6 co-analysis

7. MBNMAdose-based Hellinger distance and MCMC diagnostics

8. Data preprocessing and reproducibility note
9. IL-6 influence analysis excluding Ali Ismail 2025

**Contents**

S1. Search strategy

Supplementary Table S3. Inter-rater agreement for full-text eligibility assessment

S2. Characteristics of included studies

S3. Risk of bias assessment

S4. Bayesian network meta-analysis results, GRADE assessment, and sensitivity analyses S5. Model-based dose-response network meta-analysis

S6. Exploratory co-analysis of CRP and IL-6

S7. MBNMAdose-based Hellinger distance and MCMC diagnostics

S8. Data preprocessing and reproducibility note
S9. IL-6 influence analysis excluding Ali Ismail 2025

**S1. Search Strategy**

Database search strategies and search yields.

***Supplementary file 1: Search strategy***

| Search  terms  (Pubmed ) | ( "Aged" [Mesh] OR "Aged, 80 and over" [Mesh] OR elderly[tiab] OR "older adults" [tiab] OR "older people" [tiab] OR seniors[tiab] OR aging[tiab] OR ageing[tiab] OR geriatric*[tiab] OR "older population" [tiab] )  AND ( "Exercise" [Mesh] OR "Exercise Therapy" [Mesh] OR "Resistance Training" [Mesh] OR "Physical Exertion" [Mesh] OR exercise[tiab] OR "physical activity" [tiab] OR training[tiab] OR aerobic[tiab] OR endurance[tiab] OR "resistance training" [tiab] OR "strength training" [tiab] OR "weight training" [tiab] OR walking[tiab] OR cycling[tiab] OR "tai chi" [tiab] OR yoga[tiab] OR "combined training" [tiab] )  AND ( "C-Reactive Protein" [Mesh] OR "Interleukin-6" [Mesh] OR CRP[tiab] OR "C reactive protein" [tiab] OR IL-6[tiab] OR "interleukin 6" [tiab] )  AND ( "Randomized Controlled Trial" [Publication Type] OR randomized[tiab] OR randomised[tiab] OR randomly[tiab] OR RCT[tiab] ) |
| --- | --- |
| web of science  (4516) |  |
| Scoups(3 626) |  |

Cochrane library(10 38)

Ebsco

(1642)

| Pubmed  (992) |  |
| --- | --- |
| Embase  (1886) |  |

**Supplementary Table S3. Screening Agreement**

Full-text eligibility agreement before consensus discussion.

Supplementary Materials | Template-organized version

**Supplementary Table S3. Inter-rater agreement for full-text eligibility assessment**

Cohen's kappa was calculated among the 185 full-text articles that were successfully retrieved and independently assessed by two reviewers before consensus discussion. Five reports sought for retrieval could not be obtained and were recorded	separately.

|  | Reviewer B: Include | Reviewer B: Exclude | Total |
| --- | --- | --- | --- |
| Reviewer A: Include | 50 | 6 | 56 |
| Reviewer A: Exclude | 4 | 125 | 129 |
| Total | 54 | 131 | 185 |

| Indicator | Formula | Value |
| --- | --- | --- |
| Observed agreement (Po) | (50 + 125) / 185 | 0.946 |
| Expected agreement (Pe) | [(56 x 54) + (129 x 131)] / 185^2 | 0.582 |
| Cohen's kappa | (Po - Pe) / (1 - Pe) | 0.871 |

Interpretation: kappa = 0.871 indicates almost perfect agreement between the two independent reviewers.

**S2. Characteristics of Included Studies**

Study-level and arm-level characteristics for CRP and IL-6 outcomes.

***Supplementary file2. Characteristics ofincluded studies***

Table S1- 1. Characteristics of included studies

| study | n | Age | Unit | Health status | Before the experiment | After | After conversion |  |
| --- | --- | --- | --- | --- | --- | --- | --- | --- |
| CRP | | | | | | | | |
| Ratree 2019 | 13  13  16  12 | 66.7 ±5.8  65.6± 4.5  68 ±7.4  67.3±5.9 | hsCRP |  | 1.64±2.05  1.64 ±2.06  1.64 ±0.85  2.16 ±1.58 | 1.51±1.16  1. 16 ±0.83  0.84 ±0.53  2.08 ±1.24 |  |  |
| Cunha2019 | 25 | 71.40±5.71 | CRP |  | 2.71 ±1.17 | 2.34 ±1.02 |  |  |
|  | 23 | 69.04±4.45 |  |  | 2.73 ±1.92 | 2.83 ± 1.41 |  |  |
| Ruangthai2020 | 16  6  13 | 67.3 ±5.9  69.7 ± 6.5  66.7 ±5. 8 | hsCRP |  | 1.61±0.90  1.51±1.09  2.21 ±1.59 | 0.80±0.51  0.91±0.49  2. 11±1.19 |  |  |
| Prasertsri2019 | 6 | 67.32± 6.89 | hsCRP |  | 1.99 ±0.45 | 1.51 ±0.3 |  |  |
|  | 25 | 67.24 ±5.29 |  |  | 1.84 ±0.27 | 1.98 ±0.31 |  |  |
| Zubac2019 | 25  11 | 66.3±4.5  67.2±5.8 | CRP | Health | 2.3±1.7  1.6±1. 1 | 3.2±2. 1  1.2±0.6 |  |  |
| Byrkjeland 2016 | 12 | 63.5 ±8.0 | CRP |  | 2.36 ±0.69 | 2.04±2.56 |  |  |
|  | 61 | 63.2 ±7.2 |  |  | 2.74±0.76 | 2.09 ±0.58 |  |  |
| Rodrigues2018 | 62  10  10 | 66.0±1.75  64.0±0.75  66.0±2.25 | CRP | NA | 1.70 ±1.59–1.82  1.56 ±1.37–1.75  1.68 ±1.55–1.81 | 1.56 ±1.44–1.69  1.47 ±1.21–1.73  1.61 ±1.48–1.74 | 1.7±0.05  1.56±0. 1  1.68±0.07 | 1.56±0.06  1.47±0.13  1.61±0.07 |
| Urzi2019 | 10 | 84.4±7.7 | CRP | Health | 3.44±2.31 | 3.04±1.64 |  |  |
|  | 11 | 88.9±5.3 |  |  | 1.63±1.32 | 2. 11±1.23 |  |  |
| Timon2024 | 9  13 | 69.6±4.3  70.8± 4.3 | CRP | NA | 3.2 ±0.9  4.0±2.0 | 3.0 ±1.2  4. 1 ±1.7 |  |  |
| Azamian2022 | 14 | 73.29±5.44 | hs-CRP |  | 1714.86 ±508.33 | 1175 ±380 | 1.71 ±0..51 | 1. 18 ±0.38 |
|  | 14 | 74.79 ±3.87 |  |  | 1895.79 ±727.69 | 1876.8 ±528.2 | 1.90 ±0.73 | 1.88 ±0.53 |

| Schober2016 | 14  30 | 84.4±5.38  84.5±7.00 | hs-CRP | NA | 1.9 ±0.3–7.6  2.0±0.5–4.8 | 2.0 ±0.6–9.6  1.8 ±0.3–8.5 | 1.85±1.57  1.94±1.57 | 1.98±1.87  1.88±1.72 |
| --- | --- | --- | --- | --- | --- | --- | --- | --- |
| Gargallo2024 | 32 | 67.24 ±4.42 | CRP |  | 3. 12 ±3.97 | 2.87±2.37 |  |  |
|  | 22 | 69.30 ±4.96 |  |  | 4. 14 ±4.87 | 2.59 ±2.19 |  |  |
|  | 25 | 68.00 ±5.01 |  |  | 2.72 ±2.02 | 3.01 ±4.34 |  |  |
| Vahlberg2017 | 25  21 | 72.7±5.5  73.7±5.4 | CRP |  | 1.4 ±2.0  3.0 ±6.2 | 1. 1 ±2.6  2.9 ±6.5 |  |  |
| Morawin2021 | 23 | 70.5±5.8 | hs-CRP |  | 2.0 ± 1.7 | 2.9 ± 1.8 |  |  |
|  | 21 | 70.5±5.8 |  |  | 1.7 ± 1.3 | 2.3 ± 1.8 |  |  |
| Luk2012 | 32  32 | 67.7±9.0  66.6± 7.9 | hsCRP |  | 1.24± 2.20  1.47± 2.26 | −0.31± 2.55 0.59± 4.47 |  |  |
| Alghadir2016 | 50 | 66.8± 3.7 | hsCRP | Health | 6.8 ± 2.5 | 2.8 ± 1.3 |  |  |
|  | 50 | 67.3± 2.8 |  |  | 2.6 ± 2. 1 | 1.8 ± 0.98 |  |  |
| Mendoza2018 | 37  48 | 67.4±4.7  68.2±6.6 | CRP |  | 3.60 ± 0.40  2.10 ± 0.50 | 3.90 ± 0.40  2.30 ± 0.30 |  |  |
| Tew2012 | 11 | 71±8 | hs-CRP |  | 1.4±1. 1 | 0.9±0.9 |  |  |
|  | 14 | 74±6 |  |  | 2.3±1.5 | 2.6±1.6 |  |  |
| Jensen2019 | 23  21 | 69.9±7.5  71.4±7.3 |  |  | 1. 1 ±0.3  1.2 ±0.4 | 1. 1 ±0.3  1.3 ±0.6 |  |  |
| Pandey2023 | 30 | 62.4± 10.3 | hs-CRP |  | 3.0 ± 0.9 | 2. 1 ± 0.6 |  |  |
|  | 30 | 63.7± 13.2 |  |  | 2.3 ± 0.4 | 1.8 ± 0.6 |  |  |
| Liu2026 | 44  45 | 74±6  75±6 | CRP |  | −0.33 ±−3.31, 2.64  0.91 ±−3.85, 5.66 | −2.61±−5.60, 0.38 2.04 ±−2.75, 6.82 | -0.33 ± 20. 12  0.91 ± 32.55 | -2.61± 20.25  2.04 ± 32.74 |
| Brooks2007 | 31 | 66±2 | CRP |  | 3.5 ±9.1 | 2.8 ±2.8 |  |  |
|  | 31 | 66±1 |  |  | 3.5 ±8.4 | 4. 1 ±8. 1 |  |  |
| IL-6 | | | | | | | | |
| Sardeli2022 | 23  23 | 65.4 ±4.3  65.2±4.6 | 3.92 ±5.64  3.53 ±3.23 | | | 3.44 ±4.85  3.47 ±2.56 | | |
| Tomeleri2018 | 24 | 71.0± 5.4 | 2.8 ±0.9 | | | 2.3 ±0.3 | | |
|  | 22 | 68.8 ±4.6 | 3.3 ±1.3 | | | 3.5 ±1.2 | | |
| Ali Ismail2025 | 25 | 70. 12 ±3.44 | 19. 12±7.69 | | | 5.04 ±2.58 | | |

|  | 25 | 69.84 ±3.30 |  | 20.18 ±7.42 | 21.38 ±9.06 |
| --- | --- | --- | --- | --- | --- |
| Jin2015 | 8 | 74.27± 0.62 |  | 45.92 ±1.87 | 55.39 ±2.01 |
|  | 8 | 76. 11±2.01 |  | 47.43 ±2.45 | 45.49 ±2.98 |
| Park2024 | 15 | 67.83±1.03 |  | 3.76 ±0.42 | 3. 12±0.25 |
|  | 15 | 68.08±0.90 |  | 3.75 ±0.30 | 3.68±0.31 |
| BiesekS2021 | 18 | 71.2±4.2 |  | 2.5 ±1.1 | 4.0 ±3.7 |
|  | 18 | 70.4±3.9 |  | 2.8 ±2.3 | 3. 1 ±1.2 |
| Saez2025 | 21 | 69. 10±5.01 | NA | 12.85 ±1.78 | 11.08 ±1.60 |
|  | 13 | 69.50±4.00 |  | 13.44 ±3.81 | 10.77 ±2.07 |
|  | 10 | 69.50±4.70 |  | 14.32 ±1.94 | 12.45 ±1.24 |
|  | 17 | 69.55±4.73 |  | 10.23 ±3.08 | 15.43 ±3.18 |
| Lustosa2013 | 32 | 72±4 |  | 2.5 ±1.5 | 2.4 ±2.2 |
|  | 16 | 72±4 |  | 3.0 ±2.3 | 2.9±1.7 |
| Park2025 | 20 | 77.2±5.4 | Health | 2.02±0.85 | 1.38±0.38 |
|  | 20 | 77.2±5.4 |  | 1.80±0.40 | 1.82±0.40 |
| Heo2024 | 20 | 67.95±5.05 |  | 1.88 ±0.64 | 1.42 ±0.51 |
|  | 20 | 67.45 ±4.70 |  | 1.84 ±0.70 | 1.46 ±0.33 |
|  | 21 | 67.81±4.85 |  | 1.90 ±0.59 | 1.60±0.41 |
|  | 20 | 67.30±3.45 |  | 1.82 ±0.43 | 2.34±0.56 |
| Bruunsgaard2004 | 10 | 88.6±2.25 | Health | 3.4±1.2 | 2.8±2.6 |
|  | 11 | 90.6±2.25 |  | 3.2±1. 1 | 4.4±3.4 |
| Kwok2025 | 52 | 66.9±7.9 |  | 3.38±2.86-3.89 | 2.93±2.37-3.49 3.38 ± 0.38 2.93 ± 0.41 |
|  | 54 | 63.3±7.5 |  | 3.28±2.78-3.77 | 4.04±3.47-4.61 3.28 ± 0.36 4.04 ± 0.42 |
| Park2020 | 10 | 69. 1±0.9 |  | 3.8 ±0.3 | 3.6 ±0.3 |
|  | 10 | 68.5±0.9 |  | 3.6 ±0.4 | 3.8 ±0.2 |
| Coletta2021 | 12 | 64.4±6.2 |  | 7.07± 5.61 | 6. 14± 5.29 |
|  | 9 | 66. 1±13.6 |  | 5. 16± 4.60 | 4.37± 3.53 |
|  | 11 | 62.6±7.0 |  | 7.51± 7.73 | 6. 17± 7.01 |
| Liu2024 | 16 | 60.6±55.7 |  | 5.27±0.72 | 3.38±0.57 |
|  | 16 | 61.4±3.4 |  | 5.2±0.9 | 2.8±0.6 |
|  | 16 | 61.3±4.9 |  | 5.40±0.6 | 5. 10±0.6 |

Table S1-2. Characteristics of included studies （CRPAND IL-6）

| study | n | Age | Unit | Health  status | **CRP**  Before the  experiment | **CRP**  After | **IL-6**  Before the  experiment | **IL-6**  After | **CRP**  After conversion | **IL-6**  After conversion |
| --- | --- | --- | --- | --- | --- | --- | --- | --- | --- | --- |
| Sadjapong2020 | 32  32 | 76.68±1. 14  78.87±1.32 | CRP |  | 10.15 ±6.25  11.93±17.56 | 8.16 ±8.58  11.04 ±8.93 | 3.83±5.13  3.97±5.82 | 2.49 ±4.46  4.60±6.91 |  |  |
| Tomeleri2016 | 18 | 66.8 ±3.2 | CRP | Health | 2.4 ±1.2 | 1.8 ±1.4 | 4.0 ±1.9 | 3.3 ±1.2 |  |  |
|  | 17 | 69.5±4.7 |  |  | 2.6±1.7 | 3.2±1.8 | 3.5 ±1.4 | 3.8±1.4 |  |  |
| Tait2025 | 106  99 | 77.2±6.6  77.7±7.2 | CRP | NA | 2.76±3.42  3.37±3.57 | 17.9 ±-0.4, 36. 1  4.2 ±-8.4, 16.9 | 1.85±1.67  3.06±3.61 | 5.3 ±-7.7, 18.3  -5.2 ±-21.6, 11. 1 | 17.87±9.13 4.23±6.33 | 5.3±6.63  -5.21±8.34 |
| Kim2016 | 36 | 81.4±4.3 | hs-CRP |  | 1.3 ±1.8 | 1.4 ±2.0 | 3.2 ±1.7 | 3.7±2.3 |  |  |
|  | 35 | 81.2±4.9 |  |  | 1.4 ±1.5 | 2.3±8.4 | 3. 1±1.5 | 3.6±2.1 |  |  |
| Tan2025 | 24  24 | 67.45 ±4.24  69.47±3.44 | CRP |  | 1.69±0.41  1.55±0.34 | 1.33±0.29  1.66±0.29 | 10.21 ±2.93 8.30±0.93 | 7.99±1.45  8.05±1.40 |  |  |
| Castaneda2004 | 13 | 65 ±9 | CRP |  | 7.8 ±6.0 | 6. 1 ±6.0 | 11.3 ±10.5 | 7.7 ±6.9 |  |  |
|  | 13 | 64±12 |  |  | 6.2 ±6.0 | 7.7 ±6.0 | 6.9 ±6.5 | 10.0 ±9.8 |  |  |
| Osali2020 | 11  11 | 60-65  60-65 | CRP |  | 2.24±0.67  2.29±0.71 | 1.34±0.61  2.31±0.79 | 18.05±2.34  19. 16±2.68 | 14.92±2.38  18.68±2.71 |  |  |
| Jung2022 | 14 | 75.36± 4.50 | hs-CRP |  | 1.28± 0.83 | 0.94±0.86 | 1.74±0.80 | 1.39±0.85 |  |  |
|  | 14 | 74.64 ± 5.77 |  |  | 1.24±0.81 | 1.42±0.85 | 1.71±0.88 | 1.70±0.90 |  |  |
| Wanderley2013 | 20  11  19 | 69.9 ±5.7  67.3 ± 4.9 67.8 ±5.5 | hs-CRP | Health | 0. 17±0.08–0.46  0. 16±0.07–0.39  0. 17±0.08–0.54 | 0. 14±0.07–0.25  0. 13±0.07–0.23  0.22±0.08–0.53 | 10.8±3.6–17. 1 7±4.0–16.6  6.2±2.8–27.2 | 5.2±3.3–8.4  6.4±3.3–10.6  7.8±4.5–16.6 | 2. 18±2.81 1.47±0.47 1.96±2.37 1.37±0.46  0.20±0.22 0.24±0.27 | 10.68±3.62 5.43±1.45 8. 15±3.81 6.63±2.01  9.95±7.53 8.68±3.31 |
| Jung2024 | 14 | 78. 14±3.72 | hs-CRP |  | 1. 12±0.88 | 0.80±0.78 | 2.06±1.04 | 1.55±0.87 |  |  |

|  | 14 | 78.21±3.72 |  |  | 1. 10±0.65 | 1.24±0.77 | 1.87±1.04 | 1.97±1.37 |  |  |  |  |
| --- | --- | --- | --- | --- | --- | --- | --- | --- | --- | --- | --- | --- |
| Eleuteri2013 | 11  10 | 66±2  63±2 | CRP |  | 2. 1 ±0.3–6.9  1.2 ±0.8–8.9 | 2.0 ±0.4–10  1.6 ±0.6–27.9 | 2.3 ±0.3–9.2  1.2 ±0.8–8.9 | 1.9 ±0.5–11.3  1.6 ±0.6–27.9 | 2. 1 ± 1.65  1.2 ± 2.03 | 2.0 ± 2.40  1.6 ± 6.83 | 2.3 ± 2.23  1.2 ± 2.03 | 1.9 ± 2.70  1.6 ± 6.83 |
| Redwine2020 | 18 | 63±9 | CRP |  | 3.7 ±4.8 | 4.5 ±4.4 | 1.5 ±0.8 | 2.0 ±1.4 |  |  |  |  |
|  | 15 | 65±9 |  |  | 1.6 ±1. 1 | 1.4 ±1.5 | 1.9 ±0.9 | 1.8 ±1.6 |  |  |  |  |
|  | 18 | 67±7 |  |  | 2.0 ±3.0 | 2.2 ±2.4 | 1.3 ±0.7 | 1.3 ±0.7 |  |  |  |  |
| Campbell2009 | 48  58 | 60.5±7.0  60.9±6.8 | CRP |  | 2.39±1.85-3.10  2.36±1.88-2.97 | 2.38±1.87-3.05  2.40±1.90-3.04 | 2.36±1.82-3.05  2.26±1.80-2.83 | 2.43±1.89-3.13  2.26±1.80-2.85 | 2.39 ± 0.44  2.36 ± 0.42 | 2.38 ± 0.41  2.40 ± 0.44 | 2.36 ± 0.43  2.26 ± 0.40 | 2.43 ± 0.44  2.26 ± 0.41 |
| Ryrs2018 | 15 | 63±2 | CRP |  | 2±3.0 | 2±3.0 | 0.78±0.90 | 1.05±1.25 |  |  |  |  |
|  | 15 | 63±2 |  |  | 2±6.5 | 2±4.5 | 0.8±1.22 | 1. 12±1.35 |  |  |  |  |
| Timon2021 | 17  18  19 | 68.4± 3.8  70.3± 3.3  70.5± 4.0 | CRP | NA | 7.3 ± 2.0  7.6 ± 4.2  6.9 ± 1.3 | 3.6 ± 2.2  2.2 ± 0.9  6.5 ± 2.0 | 2.5 ± 1.2  2.5 ± 1.4  2.3 ± 1.0 | 2.6 ± 1.4  2.7 ± 1.6  2.4 ± 1.2 |  |  |  |  |
| Zhou2020 | 59 | 67 ± 14 | CRP |  | 3.5±1.5–5.9 | 3±1.5–6 | 2.3±1.6–4 | 2.2±1.5–3.7 | 3.5 ± 1.72 | 3.0 ± 1.76 | 2.3 ± 0.94 | 2.2 ± 0.86 |
|  | 53 | 66 ± 13 |  |  | 2.9±1.3–5.9 | 3±1.2–5 | 1.7±1–2.8 | 2. 1±1–3.1 | 2.9 ± 1.66 | 3.0 ± 1.37 | 1.7 ± 0.65 | 2.1 ± 0.76 |
| Andrade2021 | 16  16 | 66±2  69±3 | CRP |  | 2180±220  1820±180 | 1610±190  2150±250 | 5.4±0.6  4.5±0.3 | 5. 1±0.6  4.7±0.5 | 2. 18±0.22  1.82±0.18 | 1.61±0.19  2. 15±0.25 | 5.4±0.6  4.5±0.3 | 5. 1±0.6  4.7±0.5 |

**S3. Risk of Bias Assessment**

Detailed and summary risk-of-bias figures.

***Supplementary file3. Risk of bias in the included studies***

**Figure S1.** The detail of risk of bias

**Figure S2.** The summary of risk of bias

**S4. Bayesian NMA, GRADE, and Sensitivity Analyses**

Model fit, network plots, SUCRA, consistency, GRADE, and sensitivity analyses.

**S4.2 Model fit summaries for included studies**

S4.2.1 Model fit summaries for all studies and sensitivity analyses Table S3-(CRP). Model fit summaries for all studies and sensitivity analyses

| **Model** | **Glycosylated hemoglobin (84 data points)** | | | |
| --- | --- | --- | --- | --- |
|  | **DIC** | **pD** | **Residual**  **Deviance** | **SD(95%CrI)** |
|  | **All studies (84 data points)** | | | |
| FE Model | 515.476 | 44.189 | 559.665 | - |
| RE Model | 163.533 | 78.634 | 84.898 | 2.240(1.707,2.943) |
| RE UME | 128. 1 | 82.1 | 85.862 | - |
|  | **Exclude high residual deviance (80 data points)** | | | |
| FE Model | 549.449 | 42.3179 | 507.131 | - |
| RE Model | 155.704 | 74.938 | 80.766 | 2.273(2.985,2.985) |
|  | **Exclude interventions less than 12weeks (73 data points)** | | | |
| FE Model | 489.840 | 39.281 | 450.559 |  |
| RE Model | 141.824 | 68.514 | 73.309 | 2.445(1.827,3.265) |
| **The study excluded healthy individuals (73 data points)** | | | | |
| FE Model | 433.074 | 39.185 | 393.888 |  |
| RE Model | 142.024 | 68.744 | 73.279 | 2.494(1.86,3.324) |

***Note:*** CrI, credible interval. DIC, deviance information criterion. FE, fixed effects. pD, number of effective parameters. SD, standard deviation. RE, random effects.

Table S3-(IL-6). Model fit summaries for all studies and sensitivity analyses

| **Model** | **Glycosylated hemoglobin (64 data points)** | | | |
| --- | --- | --- | --- | --- |
|  | **DIC** | **pD** | **Residual**  **Deviance** | **SD(95%CrI)** |
|  | **All studies (64 data points)** | | | |
| FE Model | 565.145 | 37.386 | 527.759 | - |
| RE Model | 133.763 | 65.154 | 68.6050 | 4.156(3.09,5.608) |
| RE UME | 136.2 | 73. 1 | 73.697 | - |
| **Exclude interventions less than 12weeks (**57 **data points)** | | | | |
| FE Model | 458.977 | 32.18259 | 426.7953 | - |
| RE Model | 112.949 | 55.215 | 57.733 | 3.880(2.838,5.334) |
| **The study excluded healthy individuals (58 data points)** | | | | |
| FE Model | 540. 142 | 33.048 | 507.093 | - |
| RE Model | 116.284 | 56.859 | 59.425 | 4.578(3.319,6.321) |

***Note:*** CrI, credible interval. DIC, deviance information criterion. FE, fixed effects. pD, number of effective parameters. SD, standard deviation. RE, random effects.

Figure S3-(CRP). Network plot of comparisons for all studies included in the network

meta-analysis. Line width is proportional to the number of pairwise effect size estimates

and node size is proportional to the number of participants.

Figure S3-(IL-6). Network plot of comparisons for all studies included in the network

meta-analysis. Line width is proportional to the number of pairwise effect size estimates

and node size is proportional to the number of participants.

S4.2.2.SUCRA of sensitivity analyses .

Figure S4-(CRP) SUCRA of sensitivity analyses. Forest plot and league table for sensitivity analyses

Figure S4-(CRPAND IL-6) Residual Deviation Results

Figure S4-(IL-6) SUCRA of sensitivity analyses. Forest plot and league table for sensitivity analyses

Table S4-(CRP). league table for sensitivity analyses

| **Exclude high residual deviance** | | | | | |
| --- | --- | --- | --- | --- | --- |
| RE |  |  |  |  |  |
| -0.02 (-1.67, 1.60) | AE |  |  |  |  |
| -0.19 (-2.81, 2.47) | -0.17 (-2.85, 2.52) | CT |  |  |  |
| -0.68 (-2.98, 1.55) | -0.66 (-2.97, 1.58) | -0.49 (-3.66, 2.67) | MBE |  |  |
| -0.70 (-1.92, 0.48) | -0.68 (-1.94, 0.58) | -0.51 (-2.98, 1.95) | -0.02 (-2.00, 2.00) | Control |  |
| -2.37 (-4.38,-0.38) | -2.35 (-4.57, -0. 16) | -2.18 (-5.24, 0.85) | - 1.69 (-4.38, 0.99) | - 1.67 (-3.49, 0. 14) | MCB |
| **Exclude interventions less than twelve weeks** | | | | | |
| AE |  |  |  |  |  |
| -0. 12 (-2.14, 1.95) | RE |  |  |  |  |
| -0.32 (-3.35, 2.74) | -0.19 (-3.13, 2.73) | CT |  |  |  |
| -0.96 (-3.96, 1.99) | -0.84 (-3.61, 1.93) | -0.64 (-4.35, 3.04) | MBE |  |  |
| -0.86 (-2.51, 0.80) | -0.73 (-2. 12, 0.63) | -0.54 (-3.25, 2. 16) | 0.10 (-2.40, 2.65) | Control |  |
| -2.08 (-4.48, 0.33) | - 1.96 (-4.02, 0.07) | - 1.77 (-5.01, 1.50) | - 1. 12 (-4.12, 1.94) | - 1.23 (-3.00, 0.53) | MCB |
| **Exclude healthy individuals from the study population** | | | | | |
| RE |  |  |  |  |  |
| -0.26 (-2.22, 1.69) | AE |  |  |  |  |
| -0.19 (-3.08, 2.73) | 0.06 (-2.86, 3.05) | CT |  |  |  |
| -0.71 (-3.17, 1.74) | -0.45 (-2.95, 2.02) | -0.51 (-3.95, 2.88) | MBE |  |  |
| -0.69 (-2.12, 0.74) | -0.43 (-1.93, 1.07) | -0.49 (-3.17, 2. 14) | 0.02 (-2. 12, 2. 15) | Control |  |
| - 1.91 (-4.06, 0.21) | - 1.65 (-4.05, 0.73) | - 1.71 (-5.01, 1.51) | - 1.20 (-4.03, 1.59) | - 1.22 (-3. 11, 0.65) | MCB |

***Note:*** AE(A), Aerobic exercise; RE(B), Resistance exercise; CT(C), Combined Training; MBE(D), Mind-Body Exercise; MCB(F), Multi-component training

Table S4-(IL-6). . league table for sensitivity analyses

| **Exclude interventions less than twelve weeks** | | | | | |
| --- | --- | --- | --- | --- | --- |
| AE |  |  |  |  |  |
| -0.94 (-5.97, 4.08) | CT |  |  |  |  |
| - 1.52 (-6.62, 3.60) | -0.58 (-5.53, 4.39) | MCB |  |  |  |
| - 1.63 (-7.67, 4.49) | -0.69 (-6.18, 4.77) | -0. 11 (-6.19, 5.93) | MBE |  |  |
| - 1.65 (-5.29, 2.00) | -0.71 (-4.19, 2.82) | -0.13 (-3.70, 3.43) | -0.02 (-4.93, 4.95) | Control |  |
| -2.10 (-6.18, 1.99) | - 1.16 (-5.17, 2.98) | -0.58 (-4.51, 3.40) | -0.47 (-5.68, 4.70) | -0.45 (-2.59, 1.69) | RE |
| **Exclude healthy individuals from the study population** | | | | | |
| MBE |  |  |  |  |  |
| - 1.95 (-7.85, 3.83) | CT |  |  |  |  |
| -2.66 (-8.54, 3. 15) | -0.71 (-6.65, 5.33) | AE |  |  |  |
| -3.02 (-9.25, 3.02) | - 1.07 (-7.25, 5. 18) | -0.36 (-6.18, 5.45) | MCB |  |  |
| -3.19 (-7.72, 1. 16) | - 1.24 (-5.80, 3.29) | -0.53 (-4.46, 3.42) | -0.18 (-4.43, 4.08) | Control |  |
| -3.47 (-8.43, 1.40) | - 1.51 (-6.69, 3.69) | -0.80 (-5.23, 3.66) | -0.45 (-5.03, 4.28) | -0.27 (-2.85, 2.32) | RE |

**S4.3 Comparison-adjusted funnel plot of all studies**

Figure S5-(CRP) AE(l), Aerobic exercise; RE(2), Resistance exercise; CT(3), Combined Training; MBE(4), Mind-Body Exercise; MCB(5), Multi-component training

Figure S5-(IL-6) AE(l), Aerobic exercise; RE(2), Resistance exercise; CT(3), Combined Training; MBE(4), Mind-Body Exercise; MCB(5), Multi-component training

Figure S6-(CRP).Contour-enhanced funnel plot

Figure S6-(IL-6).Contour-enhanced funnel plot

**S4.4 GRADE Assessment**

Table S5-(CRP). Details of GRADE assessment for all pairwise comparisons within the depression network.

| Comparison | k | Direct SMD  [95% CI] | Quality of evidence | Indirect SMD  [95% CI] | Quality of evidence | Network SMD  [95% CI] | Quality of evidence | P-value |
| --- | --- | --- | --- | --- | --- | --- | --- | --- |
| AE vs CON | 12 | -0.79 [-1.22, -0.36] | Moderate* | -0.10 [-1.23, 1.04] | Very Low*† | -0.70 (-1.10,-0.30) | Moderate* | 0.267 |
| RT vs CON | 14 | -0.48 [-0.87, -0.08] | Moderate* | -0.32 [-1.13, 0.49] | Very low*‡ | -0.45 (-0.80,-0. 10) | Moderate* | 0.732 |
| CT vs CON | 3 | -0.26 [-1.08, 0.57] | Very low*‡ | - 1.41 [-3.61, 0.80] | Very low*‡ | -0.40 (-1.17,0.37) | Very low*‡ | 0.341 |
| MBE vs CON | 5 | -0.13 [-0.78, 0.51] | Very low*‡ | - 1.15 [-3.10, 0.81] | Very low*‡ | -0.23 (-0.84,0.38) | Moderate* | 0.333 |
| MCE vs CON | 7 | 0.25 [-0.29, 0.79] | Very low*‡ | -0.41 [-1.40, 0.57] | Very low*‡ | 0.09 (-0.38,0.57) | Low*‡ | 0.247 |
| RT vs AE | 3 | -0.06 [-0.93, 0.81] | Very low*‡ | 0.41 [-0.20, 1.01] | Very low*‡ | 0.25 (-0.24,0.75) | Low*† | 0.385 |
| CT vs AE | 1 | -0.45 [-1.93, 1.04] | Very low*‡ | 0.65 [-0.37, 1.66] | Very low*‡ | 0.30 (-0.54, 1. 14) | Very low*‡ | 0.233 |
| MBE vs AE | 1 | -0.56 [-2. 11, 1.00] | Very low*‡ | 0.73 [-0.05, 1.51] | Very low*‡ | 0.47 (-0.23, 1. 17) | Very low*‡ | 0.149 |
| MCE vs AE | 0 | NA |  | NA |  | 0.79 (0.18, 1.41) | Moderate* | NA |
| CT vs RT | 1 | -0.22 [-1.73, 1.28] | Very low*‡ | 0.16 [-0.84, 1. 16] | Very low*‡ | 0.04 (-0.78,0.87) | Very low*‡ | 0.677 |
| MBE vs RT | 1 | 0.31 [-1.18, 1.79] | Very low*‡ | 0.19 [-0.59, 0.97] | Very low*‡ | 0.22 (-0.46,0.90) | Very low*‡ | 0.894 |
| MCE vs RT | 3 | 0.06 [-0.71, 0.83] | Very low*‡ | 0.91 [0.23, 1.60] | Low*† | 0.54 (0.02, 1.07) | Moderate* | 0.105 |
| MBE vs CT | 0 | NA |  | NA |  | 0.17 (-0.81, 1. 15) | Low*‡ | NA |
| MCE vs CT | 0 | NA |  | NA |  | 0.50 (-0.40, 1.40) | Very low*‡ | NA |
| MCE vs MBE | 0 | NA |  | NA |  | 0.32 (-0.44, 1.09) | Very low*‡ | NA |

k = Number of studies providing direct evidence, prop = proportion of direct evidence, NMA (SMD) = estimated treatment effect (SMD) in network meta-analysis, direct (SMD) = estimated treatment effect (SMD) derived from direct evidence, indirect (SMD) = estimated treatment effect (SMD) derived from indirect evidence, p = p-value of test for disagreement (difference between direct and indirect evidence)

Reasons for downgrading: †Imprecision (1 downgrade), ‡Severe imprecision (2 downgrades), *risk of bias (1 downgrade)

The p-value indicated that the findings between direct and indirect comparisons was not statistically significant and therefore no comparisons have been downgraded based on inconsistency

Table S5-(IL-6）. Details of GRADE assessment for all pairwise comparisons within the depression network.

| Comparison | k | Direct SMD  [95% CI] | Quality of evidence | Indirect SMD  [95% CI] | Quality of evidence | Network SMD  [95% CI] | Quality of evidence | P-value |
| --- | --- | --- | --- | --- | --- | --- | --- | --- |
| AE vs CON | 12 | -0.44 [-1.32, 0.45] | Very low*‡ | -0.55 [-2.58, 1.47] | Very Low*† | -0.45 (-1.25,0.34) | Moderate* | 0.267 |
| RT vs CON | 14 | -0.22 [-0.82, 0.38] | Very low*‡ | -0.94 [-2.43, 0.55] | Very low*‡ | -0.32 (-0.87,0.23) | Moderate* | 0.732 |
| CT vs CON | 3 | -0.76 [-1.83, 0.32] | Very low*‡ | -2.41 [-6.78, 1.96] | Very low*‡ | -0.85 (-1.88,0. 18) | Very low*‡ | 0.341 |
| MBE vs CON | 5 | - 1.29 [-2.36, -0.23] | Moderate* | -0.22 [-3.16, 2.71] | low*† | - 1.17 (-2.16,-0. 18) | Moderate* | 0.333 |
| MCE vs CON | 7 | -0.20 [-1.28, 0.87] | Very low*‡ | 0.32 [-1.83, 2.48] | Very low*‡ | -0.10 (-1.04,0.84) | Low*‡ | 0.247 |
| RT vs AE | 3 | 0.36 [-1.17, 1.89] | Very low*‡‡ | 0.01 [-1. 12, 1. 15] | Very low*‡ | 0. 14 (-0.76, 1.03) | Low*† | 0.385 |
| CT vs AE | 1 | NA |  | NA |  | -0.40 (-1.70,0.91) | Very low*‡ | 0.233 |
| MBE vs AE | 1 | NA |  | NA |  | -0.72 (-1.98,0.54) | Very low*‡ | 0.149 |
| MCE vs AE | 0 | NA |  | NA |  | 0.35 (-0.87, 1.57) | Moderate* | NA |
| CT vs RT | 1 | 0.53 [-1.53, 2.59] | Very low*‡ | - 1.35 [-2.59, -0. 11] | low*† | -0.53 (-1.70,0.63) | Very low*‡ | 0.677 |
| MBE vs RT | 1 | 0.61 [-1.47, 2.68] | Very low*‡ | 0.08 [-1. 14, 1.30] | Very low*‡ | -0.85 (-1.94,0.24) | Very low*‡ | 0.894 |
| MCE vs RT | 3 | NA |  | NA |  | 0.22 (-0.81, 1.25) | Moderate* | 0.105 |
| MBE vs CT | 0 | 0.18 [-1.97, 2.32] | Very low*‡ | -0.63 [-2.34, 1.08] | Very low*‡ | -0.32 (-1.64, 1.00) | Low*‡ | NA |
| MCE vs CT | 0 | NA |  | NA |  | 0.75 (-0.65,2. 15) | Very low*‡ | NA |
| MCE vs MBE | 0 | NA |  | NA |  | 1.07 (-0.29,2.43) | Very low*‡ | NA |

k = Number of studies providing direct evidence, prop = proportion of direct evidence, NMA (SMD) = estimated treatment effect (SMD) in network meta-analysis, direct (SMD) = estimated treatment effect (SMD) derived from direct evidence, indirect (SMD) = estimated treatment effect (SMD) derived from indirect evidence, p = p-value of test for disagreement (difference between direct and indirect evidence)

Reasons for downgrading: †Imprecision (1 downgrade), ‡Severe imprecision (2 downgrades), *risk of bias (1 downgrade)

The p-value indicated that the findings between direct and indirect comparisons was not statistically significant and therefore no comparisons have been downgraded based on inconsistency

**S4.5 Sensitivity analysis**

Figure S7-(CRP)**.** Forest plot of sensitivity analyses.

Figure S7-(IL-6). Forest plot of sensitivity analyses.

Figure S8-(CRP). Direct comparisons in the network

Figure S8-(IL-6). Direct comparisons in the network

Figure S9-(CRP)Cumulative Probability

Figure S9-(IL-6)Cumulative Probability

**S5. Model-Based Dose-Response Network Meta-Analysis**

Dose datasets, dose networks, consistency, model selection, rankings, fit plots, and meta-regression.

***Supplementary file5. Dose network meta- analysis supplementary Online Content***

**S5.1 Table S6. Dataset**

This supplementary file shows the datasets (i.e., at global exercise and agent levels) used in this study. The studyID indicates the name of the author and the year of publication. The y indicates the mean change from the baseline of the evaluation tool. The se indicates the standard error of the mean. The agent indicates the agent level. The Mets session indicates the energy expenditure in a session of the study. The exact_dose parameter indicates the exact estimated METs per week that participants accumulated in the study. The dose indicates the group of doses by approximation. The residual_dose indicates the difference between the exact dose and the dose allocated by approximation. The frequency is the number of days those participants were involved in physical activity. The min_day indicates how many minutes a session lasted. The N indicates the number of participants.

Table S6-(CRP). Dataset

| **study** | **type** | **mean** | **sd** | **SE** | **Frequency** | **Time/session** | **Exact dose** | **Dose** | **Residual dose** |
| --- | --- | --- | --- | --- | --- | --- | --- | --- | --- |
| Ratree2019 | AE RT  CT  CON | -0.13  -0.48  -0.8  -0.08 | 1.78  1.8  0.74  1.44 | 0.4937  0.4992  0.185  0.4157 | 3  3  3 | 60  60  60 | 648  792  660 | 600  900  600  0 | 48  108  60 |
| Cunha2019 | RT | -0.37 | 1. 1 | 0.22 | 3 | 20 | 180 | 300 | 120 |
|  | CON | 0. 1 | 1.72 | 0.3586 |  |  |  | 0 |  |
| Ruangthai 2020 | CT  CT  CON | -0.81  -0.6  -0. 1 | 0.78  0.95  1.43 | 0.195  0.2635  0.4128 | 3  3 | 60  60 | 720  779.4 | 600  900 | 120  120.6  0 |
| Prasertsri 2019 | AE | -0.48 | 0.4 | 0.08 | 3 | 30 | 207 | 300 | 93 |
|  | CON | 0. 14 | 0.29 | 0.058 |  |  |  | 0 | 0 |
| Zubac 2019 | MCE  CON | 0.9  -0.4 | 1.93  0.95 | 0.5819  0.2742 | 3 | 60 | 930.6 | 900  0 | 30.6  0 |
| Byrkjeland 2016 | CT | -0.32 | 2.29 | 0.2932 | 1 | 150 | 562.5 | 600 | 47.5 |
|  | CON | -0.65 | 0.69 | 0.0876 |  |  |  | 0 | 0 |

| Rodrigues2018 | MBE AE  CON | -0. 14  -0.09  -0.07 | 0.06  0. 12  0.07 | 0.019  0.0379  0.0221 | 3  3 | 60  60 | 720  540 | 600  600  0 | 120  60  138 |
| --- | --- | --- | --- | --- | --- | --- | --- | --- | --- |
| Urzi 2019 | RT | -0.4 | 2.06 | 0.6211 | 3 | 122.5 | 458.16 | 600 | 141.84 |
|  | CON | 0.48 | 1.28 | 0.4267 |  |  |  | 0 | 0 |
| Timon 2024 | MCE  CON | -0.2  0. 1 | 1.08  1.87 | 0.2995  0.5 | 3 | 30 | 382.5 | 300  0 | 82.5  0 |
| Azamian 2022 | RT | -0.53 | 0.46 | 0.1229 | 3 | 55 | 585.75 | 600 | 14.25 |
|  | CON | -0.02 | 0.65 | 0.1737 |  |  |  | 0 | 0 |
| Schober 2016 | RT  CON | 0.13  -0.06 | 1.74  1.65 | 0.3177  0.2917 | 3 | 122.5 | 392 | 300  0 | 92  0 |
| Gargallo 2024 | RT | -0.25 | 3.46 | 0.7377 | 2 | 75 | 600 | 600 | 0 |
|  | MCE | - 1.55 | 4.22 | 0.844 | 2 | 75 | 525 | 600 | 75 |
|  | CON | 0.29 | 3.76 | 0.752 |  |  |  | 0 | 0 |
| Vahlberg 2017 | MCE  CON | -0.3  -0. 1 | 2.36  6.36 | 0.5563  1.3879 | 2 | 55 | 495 | 600  0 | 105  0 |
| Morawin 2021 | MBE | 0.9 | 1.75 | 0.3649 | 2 | 40 | 280 | 300 | 20 |
|  | CON | 0.6 | 1.61 | 0.3513 |  |  |  | 0 | 0 |
| Luk 2012 | AE  CON | - 1.55  -0.88 | 2.39  3.87 | 0.4225  0.6841 | 3 | 60 | 1215 | 1200 | 15 |
| Alghadir 2016 | AE | -4 | 2.17 | 0.3069 | 3 | 60 | 630 | 600 | 30 |
|  | CON | -0.8 | 1.82 | 0.2574 |  |  |  | 0 |  |
| Mendoza 2018 | MBE  CON | 0.3  0.2 | 0.4  0.44 | 0.0658  0.0635 | 5 | 50 | 775 | 900  0 | 125  0 |
| Tew2012 | AE | -0.5 | 1.01 | 0.3045 | 3 | 40 | 840 | 900 | 60 |
|  | CON | 0.3 | 1.55 | 0.4143 |  |  |  | 0 | 0 |

| Jensen2019 | AE  CON | 0  0. 1 | 0.3  0.53 | 0.0626  0.1156 | 3 | 60 | 810 | 900  0 | 90  0 |
| --- | --- | --- | --- | --- | --- | --- | --- | --- | --- |
| Pandey2023 | MBE  CON | -0.9 -0.5 | 0.79 0.53 | 0.1442 0.0968 | 4 | 45 | 900 | 900  0 | 120  0 |
| Liu2026 | RT | -2.28 | 20.19 | 3.0438 | 6 | 60 | 1080 | 1200 | 120 |
|  | MCE | 1.13 | 32.65 | 4.8672 | 6 | 60 | 1620 | 1500 | 120 |
| Brooks2007 | RT  CON | -0.7 0.6 | 8.07 8.25 | 1.4495 1.4818 | 3 | 35 | 294 | 300  0 | 6  0 |
| Sadjapong 2020 | MCE  CON | - 1.99  -0.89 | 7.68  15.21 | 1.3576  2.6888 | 3 | 60 | 810 | 900  0 | 90  0 |
| Tomeleri 2016 | RT  CON | -0.6 0.6 | 1.31 1.75 | 0.3088 0.4244 | 3 | 47.5 | 541.5 | 600  0 | 58.5 0 |
| Tait 2025 | MCE  CON | 15. 11  0.86 | 7.99  5.5 | 0.7761  0.5528 | 2 | 105 | 367.5 | 300  0 | 67.5  0 |
| Kim H2016 | RT  CON | 0. 1  0.9 | 1.91 7.76 | 0.3183 1.3117 | 2 | 60 | 456 | 600  0 | 144  0 |
| Tan 2025 | RT  CON | -0.36  0. 11 | 0.37  0.32 | 0.0755  0.0653 | 3 | 60 | 684 | 600  0 | 84  0 |
| Castaneda 2004 | RT  CON | - 1.7 1.5 | 6  6 | 1.6641 1.6641 | 3 | 52.5 | 787.5 | 900  0 | 112.5 0 |
| Osali 2020 | AE  CON | -0.9  0.02 | 0.64  0.75 | 0.193  0.2261 | 3 | 53.5 | 255.73 | 300  0 | 44.27  0 |
| Jung 2022 | MCE  CON | -0.34 0.18 | 0.85 0.83 | 0.2272 0.2218 | 3 | 60 | 1105.2 | 1200  0 | 94.8 0 |
| Wanderley 2013 | AE | -0.71 | 2.61 | 0.5836 | 3 | 50 | 517.5 | 600 | 82.5 |
|  | RT | -0 59 | 2 18 | 0 6573 | 3 | 50 | 562 5 | 600 | 38 5 |

CON	0.04	0.25	0.0574	0	0

| Jung 2024 | MCE | -0.32 | 0.83 | 0.2218 | NA | 187.5 | 1218.75 | 1200 | 18.75 |
| --- | --- | --- | --- | --- | --- | --- | --- | --- | --- |
|  | CON | 0. 14 | 0.72 | 0.1924 |  |  |  | 0 | 0 |
| Eleuteri 2013 | AE  CON | -0. 1  0.4 | 2.13  6.07 | 0.6422  1.9195 | 5 | 5 | 1000 | 900  0 | 100  0 |
| Redwine 2020 | MBE | 0.8 | 4.61 | 1.0866 | NA | 195 | 838.5 | 900 | 61.5 |
|  | RT | -0.2 | 1.35 | 0.3486 | NA | 195 | 624 | 600 | 24 |
|  | CON | 0.2 | 2.75 | 0.6482 |  |  |  | 0 | 0 |
| Campbell2009 | AE  CON | -0.01  0.04 | 0.43  0.43 | 0.0621  0.0565 | 5 | 45 | 1350 | 1200  0 | 150  0 |
| Ryrso2018 | RT | 0 | 3 | 0.7746 | 3 | 35 | 682.5 | 600 | 82.5 |
|  | AE | 0 | 5.77 | 1.4898 | 3 | 35 | 630 | 600 | 30 |
| Timon2021 | RT  RT  CON | -3.7  -5.4  -0.4 | 2. 11  3.83  1.76 | 0.5118  0.9027  0.4038 | 3  3 | 45  45 | 810  945 | 900  900  0 | 90  45  0 |
| Zhou 2020 | RT | -0.5 | 1.74 | 0.2265 | NA | 150 | 870 | 900 | 30 |
|  | MCE | 0. 1 | 1.54 | 0.2115 | NA | 150 | 600 | 600 | 0 |
| Andrade2021 | AE  CON | -0.57  0.33 | 0.21  0.22 | 0.0525  0.055 | 2 | 60 | 324 | 300  0 | 24  0 |

Table S6-(IL-6). Dataset

| study | type | mean | sd | SE | Frequency | Time/session | Exact dose | Dose | Residual dose |
| --- | --- | --- | --- | --- | --- | --- | --- | --- | --- |
| Sardeli 2022 | CT  CON | -0.48  -0.06 | 5.29  2.95 | 1.103  0.6151 | 2 | 90 | 793.8 | 900  0 | 106.2  0 |
| Tomeleri 2018 | RT | -0.5 | 0.79 | 0.1613 | 3 | 47.5 | 483.3 | 600 | 116.7 |
|  | CON | 0.2 | 1.25 | 0.2665 |  |  |  | 0 | 0 |
| Ali Ismail 2025 | MBE  CON | - 14.08  1.2 | 6.78  8.36 | 1.356  1.672 | 5 | 60 | 825 | 900  0 | 75  0 |
| Jin 2015 | RT | 9.47 | 1.94 | 0.6859 | 3 | 60 | 570.6 | 600 | 29.4 |
|  | CON | - 1.94 | 2.75 | 0.9723 |  |  |  | 0 | 0 |
| Park 2024 | CT  CON | -0.64  -0.07 | 0.37  0.31 | 0.0955  0.08 | 3 | 120 | 1425.6 | 1500  0 | 74.4  0 |
| Biesek S2021 | MCE | 1.5 | 3.29 | 0.7755 | 2 | 50 | 320 | 300 | 20 |
|  | CON | 0.3 | 1.99 | 0.469 |  |  |  | 0 | 0 |
| Saez 2025 | RT  RT  RT  CON | - 1.77  -2.67  - 1.87  5.2 | 1.7  3.3  1.7  3.13 | 0.371  0.9153  0.5376  0.7591 | 3  3  3 | 60  60  60 | 689.4  750.6  630 | 600  900  600  0 | 89.4  149.4  30  0 |
| Lustosa 2013 | RT | -0. 1 | 1.95 | 0.3447 | 3 | 60 | 750.6 | 900 | 149.4 |
|  | CON | -0.1 | 2.07 | 0.5175 |  |  |  | 0 | 0 |
| Park 2025 | CT  CON | -0.64  0.02 | 0.74  0.4 | 0.1655  0.0894 | 3 | 50 | 465 | 600  0 | 135  0 |
| Heo 2024 | RT | -0.46 | 0.59 | 0.1319 | 3 | 50 | 900 | 900 | 0 |
|  | RT | -0.38 | 0.61 | 0.1364 | 3 | 50 | 525 | 600 | 75 |
|  | RT  CON | -0.3 0.52 | 0.52 0.51 | 0.1135 0.114 | 3 | 50 | 420 | 300  0 | 120  0 |

| Bruunsgaard 2004 | RT  CON | -0.6  1.2 | 2.25  3 | 0.7115  0.9045 | 3 | 45 | 830 | 900  0 | 70  0 |
| --- | --- | --- | --- | --- | --- | --- | --- | --- | --- |
| Kwok 2025 | MBE | -0.45 | 0.4 | 0.0555 | 1 | 90 | 202.5 | 300 | 97.5 |
|  | CON | 0.76 | 0.39 | 0.0531 |  |  |  | 0 | 0 |
| Park 2020 | CT  CON | -0.2  0.2 | 0.3  0.35 | 0.0949  0.1107 | 3 | 105 | 1575 | 1500  0 | 75  0 |
| Coletta 2021 | AE | -0.93 | 5.46 | 1.5762 | 3 | 33 | 864 | 900 | 36 |
|  | AE | -0.79 | 4.17 | 1.39 | 3 | 41 | 615 | 600 | 15 |
| Liu 2024 | CON  MBE CT  CON | - 1.34  - 1.89  -2.4  -0.3 | 7.4  0.66  0.79  4.83 | 2.2312  0.165  0.1975  1.2075 | 6  6 | 60  60 | 1080  1620 | 0  1200  1500  0 | 0  120  120  0 |
| Sadjapong 2020 | MCE | - 1.34 | 4.83 | 0.8538 | 3 | 60 | 810 | 900 | 90 |
|  | CON | 0.63 | 6.43 | 1.1367 |  |  |  | 0 | 0 |
| Tomeleri 2016 | RT  CON | -0.7  0.3 | 1.66  1.4 | 0.3913  0.3396 | 3 | 47.5 | 541.5 | 600  0 | 58.5  0 |
| Tait 2025 | RT | 3.45 | 5.97 | 0.5745 | 2 | 105 | 367.5 | 300 | 67.5 |
|  | CON | -8.27 | 7.24 | 0.7134 |  |  |  | 0 | 0 |
| Kim 2016 | RT  CON | 0.5  0.5 | 2.07  1.87 | 0.345  0.3161 | 2 | 60 | 456 | 600  0 | 144  0 |
| Tan 2025 | RT | -2.22 | 2.54 | 0.5185 | 3 | 60 | 684 | 600 | 84 |
|  | CON | -0.25 | 1.23 | 0.2511 |  |  |  | 0 | 0 |
| Castaneda 2004 | RT  CON | -3.6  3. 1 | 9.24  8.64 | 2.5627  2.3963 | 3 | 52.5 | 787.5 | 900  0 | 112.5  0 |
| Osali 2020 | AE | -3.13 | 2.36 | 0.7116 | 3 | 53.5 | 255.73 | 300 | 44.27 |
|  | CON | -0.48 | 2.7 | 0.8141 |  |  |  | 0 | 0 |

| Jung 2022 | MCE | -0.35 | 0.83 | 0.2218 | 3 | 60 | 1105.2 | 1200 | 94.8 |
| --- | --- | --- | --- | --- | --- | --- | --- | --- | --- |
|  | CON | -0.01 | 0.89 | 0.2379 |  |  |  | 0 | 0 |
| Wanderley 2013 | AE | -5.25 | 3.16 | 0.7066 | 3 | 50 | 517.5 | 600 | 82.5 |
|  | RT | - 1.52 | 3.3 | 0.995 | 3 | 50 | 562.5 | 600 | 38.5 |
| Jung 2024 | CON  MCE  CON | - 1.27  -0.51  0. 1 | 6.54  0.97  1.24 | 1.5004  0.2592  0.3314 | NA | 187.5 | 1218.75 | 0  1200  0 | 0  18.75  0 |
| Eleuteri 2013 | AE | -0.4 | 2.5 | 0.7538 | 5 | 5 | 1000 | 900 | 0 |
|  | CON | 0.4 | 6.07 | 1.9195 |  |  |  | 0 | 0 |
| Redwine 2020 | MBE RT  CON | 0.5  -0. 1  0 | 1.22  1.39  0.7 | 0.2876  0.3589  0.165 | NA  NA | 195  195 | 838.5  624 | 900  600  0 | 0  24  0 |
| Campbell 2009 | AE | 0.07 | 0.44 | 0.0635 | 5 | 45 | 1350 | 1200 | 150 |
|  | CON | 0 | 0.41 | 0.0538 |  |  |  | 0 | 0 |
| Ryrso 2018 | RT  AE | 0.27  0.32 | 1. 12  1.29 | 0.2892  0.3331 | 3  3 | 35  35 | 682.5  630 | 600  600 | 0  30 |
| Timon 2021 | RT | 0. 1 | 1.31 | 0.5118 | 3 | 45 | 810 | 900 | 90 |
|  | RT | 0.2 | 1.51 | 0.3559 | 3 | 45 | 945 | 900 | 45 |
|  | CON | 0. 1 | 1. 11 | 0.2547 |  |  |  | 0 | 0 |
| Zhou 2020 | RT  MCE | -0. 1  0.4 | 0.9  0.71 | 0.1172  0.0975 | NA  NA | 150  150 | 870  600 | 900  600 | 30  0 |
| Andrade 2021 | AE | -0.3 | 0.6 | 0.15 | 2 | 60 | 324 | 300 | 24 |
|  | CON | 0.2 | 0.44 | 0.11 | 2 | 30 | 138 | 0 | 138 |

**S5.2 Select Model Fit**

S5.2. 1 Number of original studies at different dose scenarios(CRPAND IL-6)

Figure S10-(IL-6).The dose-response relationship between overall physical activity in older adults and changes in C-reactive protein (CRP).

Figure S10-(IL-6)..The dose-response relationship between overall physical activity in older adults and changes in C-reactive protein (CRP).

Figure S11-(CRP). Number of original studies at different dose scenarios. AE(1), Aerobic exercise; RE(2), Resistance exercise; CT(3), Combined Training; MBE(4), Mind-Body Exercise; MCB(5),

Multi-component training

Figure S11-(IL-6). Number of original studies at different dose scenarios. AE(1), Aerobic

exercise; RE(2), Resistance exercise; CT(3), Combined Training; MBE(4), Mind-Body Exercise; MCB(5), Multi-component training

S5.2.2 Effect sizes ofthe original study in different types of training situations

Figure S12-(CRP). Effect sizes ofthe original study in different types of training situations. AE(1), Aerobic exercise; RE(2), Resistance exercise; CT(3), Combined Training; MBE(4), Mind-Body

Exercise; MCB(5), Multi-component training

Figure S12-(IL-6). Effect sizes ofthe original study in different types of training situations. AE(1), Aerobic exercise; RE(2), Resistance exercise; CT(3), Combined Training; MBE(4), Mind-Body

Exercise; MCB(5), Multi-component training

**S5.3 Key assumptions of Network Meta-Analysis**

The following are the three key assumptions of the network meta-analysis.

S5.3.1 Connectivity

Connectivity is a key assumption in NMA that, if considered insufficient (i.e., due to lack of direct comparisons), may lead to low statistical power and misleading results. Our study assessed network connectivity at the motor and dose levels and did not find any evidence of network unconnectedness (Figure S1 and Figure S2).

Figure S13-(CRP). Treatment-level network. The first value indicates the specific intervention and the second one is the corresponding dose of that intervention. AE(1), Aerobic exercise; RE(2),

Resistance exercise; CT(3), Combined Training; MBE(4), Mind-Body Exercise; MCB(5), Multi-component training

Figure S13-(IL-6). Treatment-level network. The first value indicates the specific intervention and the second one is the corresponding dose of that intervention. AE(1), Aerobic exercise; RE(2),

Resistance exercise; CT(3), Combined Training; MBE(4), Mind-Body Exercise; MCB(5), Multi-component training.

Figure S14-(CRP). Agent-level network. AE(1), Aerobic exercise; RE(2), Resistance exercise; CT(3), Combined Training; MBE(4), Mind-Body Exercise; MCB(5), Multi-component training

Figure S14-(IL-6). Agent-level network. AE(1), Aerobic exercise; RE(2), Resistance exercise; CT(3), Combined Training; MBE(4), Mind-Body Exercise; MCB(5), Multi-component training

S5.3.2 Consistency

We performed a consistency analysis of the data by comparing the consistency of the network (i.e., network effect size) with the unrelated mean effects (UME) model (i.e., pairwise effect sizes). In practice, we checked whether the bias, the number of estimated parameters in the network, and the Deviance Information Criterion (DIC) metric were similar for both models, indicating a good fit (47). The comparison of these parameters showed a good agreement between the different models (Table S1).

Table S7-(CRP). Consistent and UME models fit comparison

| **Model** | **pD** | **Deviance** | **Residual deviance** | **DIC** |
| --- | --- | --- | --- | --- |
| Consistent | 87. 1 | 45.839 | 85.740 | 133.0 |
| UME | 82. 1 | 45.962 | 85.862 | 128. 1 |

*Note.* pD: Number of estimated parameters; DIC: Deviance Informative Criterion; SD: Standard

Deviation; UME: Unrelated Mean Effects. Scientific literature indicated that the main indicator to assess the model fit is the DIC. As lower DIC, better fit.

Table S7-(IL-6). Consistent and UME models fit comparison

| **Model** | **pD** | **Deviance** | **Residual deviance** | **DIC** |
| --- | --- | --- | --- | --- |
| Consistent | 75.0 | 63.087 | 73.714 | 135 |
| UME | 73. 1 | 63.070 | 73.697 | 133 |

*Note.* pD: Number of estimated parameters; DIC: Deviance Informative Criterion; SD: Standard Deviation; UME: Unrelated Mean Effects. Scientific literature indicated that the main indicator to assess the model fit is the DIC. As lower DIC, better fit.

S5.2.3 Transitivity

NMAs are based on the assumption of indirect/mixed comparisons, which implies that estimates of treatment effects from direct and indirect evidence are consistent but with the usual variation of meta-analyses under a random effects model (48). This assumption is equivalent to heterogeneity in a "standard" meta-analysis (49). Following a previous proposal (50), anomalies were assessed at a deeper network level (i.e., at the treatment level). We assessed the span by the MBNMA node splitting method. This method splits the contribution of a specific treatment contrast into direct and indirect evidence and compares them (51). Similar effects indicate good span. **Table S2** presents the results for transitivity in this meta-analysis.

Table S8-(CRP). Node-splitting analysis of inconsistency

| Comparison | p-value | Median | 2.50% | 97.50% |
| --- | --- | --- | --- | --- |
| 6_600 vs 3_900 | 0.585 |  |  |  |
| -> direct |  | 0.368 | - 1.399 | 2.166 |
| -> indirect |  | 0.631 | -0. 144 | 1.345 |
| -> MBNMA |  | 0.604 | -0.046 | 1.271 |
| 6_600 vs 3_600 | 0.496 |  |  |  |
| -> direct |  | -0.101 | - 1.344 | 1.073 |
| -> indirect |  | 0.454 | -0.169 | 1.069 |
| -> MBNMA |  | 0.367 | -0.137 | 0.919 |
| 3_600 vs 2_600 | 0.632 |  |  |  |
| -> direct |  | 0.043 | - 1.228 | 1.329 |
| -> indirect |  | 0.093 | -0.452 | 0.663 |
| -> MBNMA |  | 0.083 | -0.467 | 0.608 |
| 6_ 1200 vs Placebo_0 | 0.579 |  |  |  |
| -> direct |  | -0.61 | - 1.959 | 0.776 |
| -> indirect |  | 0.123 | - 1.108 | 1.306 |
| -> MBNMA |  | -0.221 | - 1.115 | 0.672 |
| 6_900 vs Placebo_0 | 0.477 |  |  |  |
| -> direct |  | 0.342 | -0.958 | 1.661 |
| -> indirect |  | -0.363 | - 1.158 | 0.411 |
| -> MBNMA |  | -0.166 | -0.836 | 0.504 |
| 6_300 vs Placebo_0 | 0.089 |  |  |  |
| -> direct |  | 1.033 | -0.227 | 2.257 |
| -> indirect |  | -0.09 | -0.327 | 0.129 |
| -> MBNMA |  | -0.055 | -0.279 | 0.168 |
| 5_900 vs Placebo_0 | 0.274 |  |  |  |
| -> direct |  | -0.282 | - 1.151 | 0.543 |
| -> indirect |  | 0.617 | -5.151 | 5.977 |
| -> MBNMA |  | -0.318 | - 1.172 | 0.534 |
| 5_300 vs Placebo_0 | 0.287 |  |  |  |
| -> direct |  | 0.188 | - 1.69 | 1.896 |
| -> indirect |  | -0.113 | -0.398 | 0.19 |
| -> MBNMA |  | -0.106 | -0.391 | 0.178 |
| 4_900 vs Placebo_0 | 0.868 |  |  |  |
| -> direct |  | -0.462 | -2.162 | 1.28 |
| -> indirect |  | -0.672 | -2.126 | 0.834 |
| -> MBNMA |  | -0.537 | - 1.781 | 0.712 |
| 4_600 vs Placebo_0 | 0.727 |  |  |  |
| -> direct |  | -0.345 | - 1.329 | 0.638 |
| -> indirect |  | -0.106 | - 1.682 | 1.551 |
| -> MBNMA |  | -0.358 | - 1.188 | 0.474 |
| 3_300 vs Placebo_0 | 0.354 |  |  |  |

| -> direct |  | -0.126 | - 1.166 | 0.881 |
| --- | --- | --- | --- | --- |
| -> indirect |  | -0.235 | -0.443 | -0.025 |
| -> MBNMA |  | -0.239 | -0.433 | -0.044 |
| 2_ 1200 vs Placebo_0 | 0.356 |  |  |  |
| -> direct |  | -0.171 | - 1.419 | 1.09 |
| -> indirect |  | - 1.218 | -2.214 | -0.199 |
| -> MBNMA |  | -0.787 | - 1.59 | 0.006 |
| 2_900 vs Placebo_0 | 0.623 |  |  |  |
| -> direct |  | -0.303 | - 1.381 | 0.798 |
| -> indirect |  | -0.726 | - 1.42 | -0.015 |
| -> MBNMA |  | -0.59 | - 1.193 | 0.004 |
| 2_300 vs Placebo_0 | 0 |  |  |  |
| -> direct |  | -2.442 | -3.282 | - 1.557 |
| -> indirect |  | -0. 12 | -0.275 | 0.041 |
| -> MBNMA |  | -0.197 | -0.398 | 0.001 |

Table S8-(IL-6). Node-splitting analysis of inconsistency

| Comparison | p-value | Median | 2.50% | 97.50% |
| --- | --- | --- | --- | --- |
| 6_600 vs 3_900 | 0.616 |  |  |  |
| -> direct |  | 0.621 | - 1.947 | 3.077 |
| -> indirect |  | 0.266 | -0.841 | 1.427 |
| -> MBNMA |  | 0.329 | -0.683 | 1.386 |
| 3_600 vs 2_600 | 0.727 |  |  |  |
| -> direct |  | 0.439 | - 1.263 | 2.212 |
| -> indirect |  | 0.227 | -0.774 | 1.239 |
| -> MBNMA |  | 0.133 | -0.753 | 1.036 |
| 6_ 1200 vs Placebo_0 | 0.789 |  |  |  |
| -> direct |  | -0.494 | -2.365 | 1.415 |
| -> indirect |  | -0.13 | -2.849 | 2.687 |
| -> MBNMA |  | -0.378 | - 1.864 | 1.137 |
| 6_900 vs Placebo_0 | 0.718 |  |  |  |
| -> direct |  | -0.361 | -2.887 | 2.219 |
| -> indirect |  | -0.254 | - 1.499 | 1.087 |
| -> MBNMA |  | -0.284 | - 1.398 | 0.853 |
| 6_300 vs Placebo_0 | 0.261 |  |  |  |
| -> direct |  | 0.451 | -2.178 | 3.061 |
| -> indirect |  | -0.107 | -0.494 | 0.283 |
| -> MBNMA |  | -0.095 | -0.466 | 0.284 |
| 5_ 1200 vs Placebo_0 | 0.726 |  |  |  |
| -> direct |  | -0.643 | -3.014 | 1.71 |
| -> indirect |  | - 1.441 | -3.703 | 0.766 |
| -> MBNMA |  | - 1.157 | -2.773 | 0.427 |
| 5_900 vs Placebo_0 | 0.808 |  |  |  |

| -> direct |  | -0.62 | -2.319 | 1.053 |
| --- | --- | --- | --- | --- |
| -> indirect |  | - 1.041 | -2.768 | 0.705 |
| -> MBNMA |  | -0.867 | -2.08 | 0.32 |
| 5_300 vs Placebo_0 | 0.027 |  |  |  |
| -> direct |  | -3.048 | -5.346 | -0.785 |
| -> indirect |  | -0.221 | -0.585 | 0.164 |
| -> MBNMA |  | -0.289 | -0.693 | 0.107 |
| 4_ 1500 vs Placebo_0 | 0.661 |  |  |  |
| -> direct |  | - 1.206 | -2.791 | 0.293 |
| -> indirect |  | - 1.343 | -4.273 | 1.619 |
| -> MBNMA |  | - 1.294 | -2.602 | 0. 124 |
| 4_900 vs Placebo_0 | 0.465 |  |  |  |
| -> direct |  | -0.163 | -2.613 | 2.396 |
| -> indirect |  | -0.809 | - 1.665 | 0.004 |
| -> MBNMA |  | -0.776 | - 1.561 | 0.075 |
| 4_600 vs Placebo_0 | 0.34 |  |  |  |
| -> direct |  | - 1. 124 | -3.691 | 1.386 |
| -> indirect |  | -0.473 | - 1.035 | 0.102 |
| -> MBNMA |  | -0.517 | - 1.041 | 0.05 |
| 3_900 vs Placebo_0 | 0.623 |  |  |  |
| -> direct |  | -0.957 | - 1.884 | 0.013 |
| -> indirect |  | -0.447 | - 1.55 | 0.687 |
| -> MBNMA |  | -0.511 | - 1.279 | 0.275 |
| 3_300 vs Placebo_0 | 0.285 |  |  |  |
| -> direct |  | 0.163 | - 1.684 | 1.963 |
| -> indirect |  | -0.174 | -0.448 | 0.096 |
| -> MBNMA |  | -0.17 | -0.426 | 0.092 |
| 2_ 1200 vs Placebo_0 | 0.669 |  |  |  |
| -> direct |  | 0.157 | -2.283 | 2.594 |
| -> indirect |  | -0.779 | -2.736 | 1.183 |
| -> MBNMA |  | -0.414 | -2.005 | 1. 11 |
| 2_900 vs Placebo_0 | 0.808 |  |  |  |
| -> direct |  | -0.052 | - 1.739 | 1.708 |
| -> indirect |  | -0.419 | - 1.745 | 1.002 |
| -> MBNMA |  | -0.311 | - 1.504 | 0.832 |
| 2_300 vs Placebo_0 | 0.257 |  |  |  |
| -> direct |  | -0.974 | -2.727 | 0.883 |
| -> indirect |  | -0.065 | -0.466 | 0.338 |
| -> MBNMA |  | -0.104 | -0.501 | 0.277 |

***Note:*** CrI, credible interval. CAE, continuous aerobic exercise. RE, resistance exercise. CE, combined aerobic and resistance exercise. TC, tai chi. HIIT, high-intensity interval training. CG, control group.

Figure S15-(CRP). Node-splitting analysis (density plot). The value of title is the corresponding dose of that agent. CrI, credible interval. AE(1), Aerobic exercise; RE(2), Resistance exercise; CT(3), Combined Training; MBE(4), Mind-Body Exercise; MCB(5), Multi-component training

Figure S15-(IL-6) Node-splitting analysis (density plot). The value of title is the corresponding dose of that agent. CrI, credible interval. AE(2), Resistance exercise; CT(3), Combined Training; MBE(4), Mind-Body Exercise; MCB(5), Multi-component training

**S5.4 Models’ selection**

S5.4.1 Non-linear functions and models fit comparison

A meta-analysis (i.e., a "split" NMA) of the different doses of physical activity as separate and unrelated treatments were performed. This step helps determine which function is more appropriate for the data and should be used in a model-based network meta-analysis (MBNMA)

(52). **Figure S13** and **Figure S14** show the different responses of each dose to overall and different types of exercise, respectively (Hedges' g).

Figure S16-(CRP)**.** “Split” NMA of overall exercise

Figure S16-(IL-6) “Split” NMA of overall exercise

Figure S17-(CRP). “Split” NMA of different exercise agents. AE, Aerobic exercise; RE, resistance exercise; ME, mix exercise; MBE, mind-body exercise.

Figure S17-(IL-6)“Split” NMA of different exercise agents. AE, Aerobic exercise; RE, resistance exercise; ME, mix exercise; MBE, mind-body exercise.

S5.4.2 Models selection

For our data, quadratic model shows the best fit and were therefore used in subsequent analyses. Table S9-(CRP). Models fit comparison

| **Model** | | | **DIC** | **SD** | **Deviance** | **Residual**  **deviance** | **pD** |
| --- | --- | --- | --- | --- | --- | --- | --- |
| Emax (random treatment effects) | | | 470.7 | NA | 425.793 | 465.693 | 44.3 |
| Restricted cubic spline (common treatment  effects; 3 knots) | | | 221.3 | NA | 167.709 | 207.610 | 53.8 |
| Restricted cubic (random treatment 3 knots) | | spline  effects; | 126.6 | 2.275(0.304 , 1.002) | 46.954 | 86.854 | 80.2 |
| Non-parametric monotonically (common  effects) | up treatment | | 126.4 | 0.829(0.116 , 1.001) | 46.233 | 86.133 | 80.8 |

*Note.* DIC = Deviance Information Criterion; SD = Between-study Standard Deviation; pD: Number of estimated parameters; NA = Not Applicable. The SD is presented as the main value and (95% Credible

Intervals).

Table S9-(IL-6). Models fit comparison

| **Model** | | | **DIC** | **SD** | **Deviance** | **Residual**  **deviance** | **pD** |
| --- | --- | --- | --- | --- | --- | --- | --- |
| Emax (random treatment effects) | | | 648.1 | NA | 611.330 | 621.957 | 37.0 |
| Restricted cubic spline (common treatment  effects; 3 knots) | | | 416.0 | NA | 366.911 | 377.538 | 49.0 |
| Restricted cubic (random treatment 3 knots) | | spline  effects; | 134.0 | 3.815(0.536 ,5.053) | 64.564 | 75.191 | 70.0 |
| Non-parametric monotonically (common  effects) | up treatment | | 133.3 | 1.277(0.183 , 1.676) | 63.627 | 74.254 | 70.5 |

**Table S9-**(IL-6)**.** Models fit comparison

In addition to the model fit index, a deviation plot showing the contribution of each data point to the residuals can also help to confirm the robustness of the model selection. The contribution of each data point to the posterior mean bias should be around 1, which indicates a good model fit (53). Deviation plots for overall **(Figure S15)** and treatment effects **(Figure S16)** confirm the

robustness of our model selection (i.e., deviations <1.5 except for a few data points in the overall exercise and stretch at 300 METs-min, which are all below a contribution of 1.5).

Figure S18-(CRP). Deviance plot at overall exercise level.

Figure S18-(IL-6). Deviance plot at overall exercise level.

Figure S19-(CRP). Deviance plots at treatment-level.

Figure S19-(IL-6). Deviance plots at treatment-level.

**S5.5 Ranking of effectiveness of interventions**

Figure S20-(CRP) shows the probability of each intervention to be ranked from worst to best (estimated after up to 4000 iterations).

Figure S20-(IL-6) shows the probability of each intervention to be ranked from worst to best (estimated after up to 4000 iterations).

In addition, we also plotted the fit further to assess the degree of fit of the model. The fit values are plotted as connecting lines, and the observations in the original dataset are plotted as points. These plots can determine if the model fits the data well for different exercises and doses of the dose-response functionll.

Figure S2l-(CRP). Fit plots at overall exercise level.

Figure S2l-(IL-6). Fit plots at overall exercise level.

Figure S22-(CRP)**.** Fit plots at agent-level

Figure S22-(IL-6). Fit plots at agent-level

**S5.6 Models fit comparison**

S5.6. 1Models fit comparison

Table S10-(CRP) Models fit comparison

| Fitting an Inconsistency Model* | | |
| --- | --- | --- |
| chi2(13) | = | 6.72 |
| Prob > chi2 | = | 0.9161 |

Table S10-(IL-6) Models fit comparison

| Fitting an Inconsistency Model* | | |
| --- | --- | --- |
| chi2 (8) | = | 6.34 |
| Prob > chi2 | = | 0.6092 |

**S5.6.2.** Predictions ranking (from best to worst)

Table S11-(CRP). Predictions ranking (from best to worst)

| Treatment | Mean | Median | 2.50% | 97.50% |
| --- | --- | --- | --- | --- |
| 2_530 | 4.96 | 3 | 1 | 18 |
| 2_670 | 5.89 | 4 | 1 | 18 |
| 2_400 | 7.27 | 6 | 2 | 21 |
| 2_800 | 9.24 | 8 | 2 | 25 |
| 3_900 | 11.2 | 9 | 1 | 35 |
| 2_270 | 11.41 | 10 | 4 | 25 |
| 3_800 | 12.4 | 11 | 2 | 29 |
| 3_700 | 14.39 | 14 | 4 | 28 |
| 2_930 | 15.53 | 14 | 4 | 36 |
| 3_600 | 16.93 | 17 | 5 | 31 |
| 4_600 | 17.36 | 15 | 2 | 42 |
| 4_500 | 17.52 | 16 | 1 | 42 |
| 4_700 | 18.23 | 16 | 3 | 43 |
| 6_ 1200 | 18.41 | 16 | 1 | 46 |
| 4_400 | 18.79 | 17 | 1 | 44 |
| 3_500 | 19.76 | 19 | 5 | 36 |
| 2_ 130 | 19.86 | 19 | 9 | 32 |
| 4_800 | 19.93 | 18 | 2 | 45 |
| 5_900 | 20.83 | 20 | 1 | 44 |
| 4_300 | 21.18 | 20 | 3 | 45 |
| 6_ 1100 | 21.52 | 21 | 2 | 45 |
| 4_900 | 22.12 | 21 | 1 | 46 |
| 3_400 | 22.86 | 22 | 7 | 40 |
| 4_200 | 24.35 | 24 | 7 | 43 |
| 5_800 | 25.19 | 26 | 5 | 41 |
| 3_300 | 26.04 | 26 | 10 | 42 |
| 6_930 | 26.74 | 27 | 7 | 43 |
| 5_700 | 27.58 | 31 | 4 | 42 |
| 4_ 100 | 28.52 | 29 | 15 | 42 |

| 3_200 | 29.01 | 29 | 14 | 44 |
| --- | --- | --- | --- | --- |
| 5_600 | 29.06 | 35 | 2 | 44 |
| 2_ 1100 | 29.07 | 30 | 6 | 45 |
| 6_800 | 29.92 | 31 | 11 | 43 |
| 5_500 | 30.53 | 38 | 1 | 46 |
| 3_ 100 | 31.55 | 32 | 20 | 43 |
| 5_400 | 32.09 | 40 | 2 | 46 |
| 6_670 | 32.32 | 34 | 12 | 45 |
| 5_300 | 32.79 | 40 | 4 | 45 |
| 5_200 | 33.41 | 39 | 7 | 44 |
| Placebo_0 | 33.7 | 34 | 23 | 44 |
| 5_ 100 | 34.21 | 37 | 16 | 42 |
| 6_530 | 34.31 | 36 | 14 | 46 |
| 6_ 130 | 35.25 | 35 | 24 | 44 |
| 6_400 | 35.55 | 37 | 17 | 46 |
| 6_270 | 35.76 | 36 | 21 | 45 |
| 2_ 1200 | 36.45 | 40 | 8 | 46 |

***Note:*** AE(1), Aerobic exercise; RE(2), Resistance exercise; CT(3), Combined Training; MBE(4), Mind-Body Exercise; MCB(5), Multi-component training

(	)	(	)

Table 11**-** IL-6	Predictions rankin	from best to worst

g

**.**

| Treatment | Mean | Median | 2.50% | 97.50% |
| --- | --- | --- | --- | --- |
| 5_670 | 6.01 | 2 | 1 | 33 |
| 5_530 | 6.36 | 2 | 1 | 35 |
| 5_800 | 7.46 | 4 | 1 | 30 |
| 5_400 | 8.65 | 5 | 2 | 37 |
| 5_930 | 10.47 | 7 | 2 | 33 |
| 5_270 | 11.86 | 8 | 4 | 37 |
| 4_ 1500 | 14.84 | 12 | 1 | 44 |
| 4_ 1300 | 14.89 | 12 | 2 | 38 |
| 4_ 1200 | 15.69 | 13 | 3 | 39 |
| 4_ 1000 | 17.59 | 14 | 1 | 42 |
| 5_ 130 | 18.1 | 17 | 7 | 38 |
| 3_900 | 18.53 | 17 | 3 | 40 |
| 2_530 | 19.37 | 18 | 2 | 44 |
| 4_830 | 19.49 | 16 | 1 | 44 |
| 2_400 | 20.25 | 19 | 3 | 44 |
| 2_670 | 20.61 | 19 | 3 | 42 |
| 5_ 1100 | 20.76 | 18 | 2 | 45 |
| 3_800 | 21.23 | 21 | 7 | 37 |
| 4_670 | 21.85 | 19 | 2 | 46 |
| 6_ 1200 | 21.96 | 19 | 1 | 46 |
| 2_270 | 22.76 | 22 | 7 | 42.03 |

| 2_800 | 23.28 | 22 | 5 | 43 |
| --- | --- | --- | --- | --- |
| 3_700 | 24.02 | 24 | 10 | 39 |
| 6_ 1100 | 24.15 | 23 | 3 | 45 |
| 4_500 | 24.39 | 22 | 4 | 45 |
| 3_600 | 26.7 | 27 | 11 | 41 |
| 4_330 | 26.79 | 27 | 7 | 44 |
| 2_930 | 27.14 | 27 | 6 | 44 |
| 2_ 130 | 27.24 | 27 | 13 | 41 |
| 6_930 | 27.27 | 28 | 5 | 44 |
| 6_800 | 28.91 | 31 | 4 | 44 |
| 3_500 | 29.11 | 30 | 11 | 43 |
| 5_ 1200 | 29.13 | 33 | 1 | 46 |
| 4_ 170 | 29.58 | 30 | 14 | 42 |
| 6_670 | 30.26 | 33 | 3 | 46 |
| 3_400 | 31.39 | 32 | 14 | 45 |
| 6_530 | 31.58 | 35 | 3 | 46 |
| 2_ 1100 | 32.24 | 37 | 5 | 45 |
| 6_400 | 32.56 | 37 | 6 | 46 |
| 3_300 | 33.17 | 34 | 16 | 45 |
| 6_270 | 33.21 | 37 | 11 | 44 |
| Placebo_0 | 33.45 | 34 | 20 | 44 |
| 6_ 130 | 33.9 | 35 | 18 | 43 |
| 3_200 | 34.01 | 35 | 20 | 44 |
| 3_ 100 | 34.15 | 35 | 22 | 43 |
| 2_ 1200 | 34.62 | 41 | 2.98 | 46 |

***Note:*** AE(1), Aerobic exercise; RE(2), Resistance exercise; CT(3), Combined Training; MBE(4), Mind-Body Exercise; MCB(5), Multi-component training

**S5.7 Dose response network meta-regression**

We assume that the effect corrections of the different agents relative to the network reference agent are exchangeable/shared across the network around a common mean value, , with the standard deviation between agents being TY^13^:

**Table S12-**(CRP)**.** Dose response network meta-regression

| **Covariate** | **Glycosylated hemoglobin** | | | | |
| --- | --- | --- | --- | --- | --- |
|  | **DIC** | **pD** | **Residual**  **Deviance** | **Shared beta**  **(meam and 95% CrI)** | **SD** |
| Age | 122.6 | 78.49 | 44.06 | -0.016(-0.055,0.033) | 1.562 (1.195,2.027) |
| Intervention  duration (weeks) | 122.5 | 78.72 | 43.73 | -0.002(-0.056,0.073) | 1.565(1.201,2.043) |

***Note:*** CrI, credible interval. BMI, body mass index. DIC, deviance information criterion; FE, fixed effects; pD, number of effective parameters; SD, standard deviation.

**Table S12-**(IL-6)**.** Dose response network meta-regression

| **Covariate** | **Glycosylated hemoglobin** | | | | |
| --- | --- | --- | --- | --- | --- |
|  | **DIC** | **pD** | **Residual**  **Deviance** | **Shared beta**  **(meam and 95% CrI)** | **SD** |
| Age | 119.749 | 65.119 | 54.630 | -0.024(-0.091,0.095 ) | 0.047(2.916) |
| Intervention  duration (weeks) | 119.630 | 65.307 | 54.322 | 0.097(-0.732, 1.075) | 1.565(2.216,3.998) |

Bivariate ranking and exploratory association analyses for CRP and IL-6.

Supplementary Materials | Template-organized version

***Supplementary file 6. The Relationship Between CRP andIL-6***

To	further	explore	the	potential	physiological	linkage	between	exercise-induced inflammatory responses, additional study-level analyses were conducted using studies that simultaneously reported both CRP and IL-6 outcomes. Random-effects meta-regression models were performed to examine the association between exercise-induced changes in IL-6	and corresponding CRP effect sizes across studies. Sensitivity analyses were additionally conducted by excluding influential studies to evaluate the robustness of the observed associations.

Figure 23: Bivariate SUCRA scatter plots showing the anti-inflammatory effects (IL-6 and CRP) of each intervention.

Figure 24: Results of a meta-regression analysis examining the relationship between exercise-induced changes in IL-6 levels and changes in CRP levels.

Figure 25: Association Between Exercise-Induced Changes in IL-6 and CRP

Figure 26. Results of the meta-regression analysis of the dose-response relationship between intervention dose and CRP effect size.

**S7. MBNMAdose-Based Hellinger Distance and MCMC Diagnostics**

Hellinger D, between-study SD, DIC comparison, trace plots, and autocorrelation plots.

Internal suggested manuscript/response text has been excluded from the formal attachment.

Supplementary Materials | Template-organized version

**Supplementary File S7. Quadratic MBNMAdose-Based Hellinger Distance and**

**MCMC Diagnostics**

**Methodological note**

Hellinger distance (D) was calculated directly from posterior samples ofthe quadratic MBNMAdose framework. For each CRP and IL-6 analysis set, common-effect and random-effects Bayesian dose-response models were [fitted using mbnma.network](fittedusingmbnma.network)() [and mbnma.run](andmbnma.run)() with a second-degree polynomial dose-response function, dpoly(degree = 2). D was calculated between

common-effect and random-effects posterior distributions of beta.1 and beta.2 parameters. Larger D values indicate greater posterior distributional divergence after allowing for between-study heterogeneity.

Each diagnostic model used three MCMC chains, 20,000 iterations, 10,000 burn-in iterations, and thinning of 10, yielding 3,000 retained posterior samples per model. Duplicate arms with the same studyID, agent, and dose were combined using inverse-

variance weighting; studies that became single-arm after this processing were excluded from the corresponding network analysis because they do not contribute comparative evidence. Rhat and effective sample sizes were recalculated from the original chain arrays using coda diagnostics.

**Supplementary Table. Quadratic MBNMAdose Hellinger distance and MCMC performance**

| **Analysis set** | **No. beta**  **parameters** | **Median D** | **D range** | **Between-**  **study SD**  **median (95%**  **CrI)** | **Rhat range** | **Minimum**  **n.eff** | **DIC common** | **DIC random** |
| --- | --- | --- | --- | --- | --- | --- | --- | --- |
| CRP_modality | 10 | 0.645 | 0.602-0.850 | 2.319 (1.759,  3.153) | 1.000-1.001 | 2876 | 522.3 | 126.0 |
| CRP_overall_d  ose_nodes | 2 | 0.816 | 0.814-0.818 | 2.309 (1.775,  3.040) | 1.000-1.000 | 3000 | 531.1 | 101.7 |
| IL6_modality | 10 | 0.809 | 0.760-0.971 | 4.101 (3.103,  5.637) | 1.000-1.001 | 2869 | 524.9 | 132.8 |
| IL6_overall_do  se_nodes | 2 | 0.811 | 0.800-0.823 | 3.812 (2.917,  5.082) | 1.001-1.001 | 2797 | 676.9 | 129.7 |

Abbreviations: CrI, credible interval; D, Hellinger distance; DIC, deviance information criterion; MBNMAdose, model-based network meta-analysis of dose-response; n.eff, effective sample size.

**MCMC diagnostic figures**

**Supplementary Figure 1. CRP modality-level quadratic MBNMAdose model.**

**Supplementary Figure 2. CRP overall exercise dose-node quadratic MBNMAdose model.**

**Supplementary Figure 3. IL-6 modality-level quadratic MBNMAdose model.**

**Supplementary Figure 4. IL-6 overall exercise dose-node quadratic MBNMAdose model.**

**S8. Data Preprocessing and Reproducibility Note**

Model-ready inputs, duplicate-arm processing, single-arm exclusions, model settings, and session information.

**Supplementary File S8. Data Preprocessing and Reproducibility Note**

**1. Model-ready datasets**

The Bayesian NMA/MBNMAdose analyses used the model-ready arm-level datasets exported from the original CRP and IL-6 spreadsheets. The required fields were studyID, agent, dose, y, se, and n. The CRP modality-level model initially contained 86 arm-level rows; the IL-6 modality-level model initially contained 73 arm-level rows.

For the overall exercise dose-node analyses, exercise arms were coded as exercise at the corresponding dose node and control arms were coded as zero-dose placebo/control arms. These datasets were used only when the network contained valid comparative information within each study.

**2. Duplicate-arm processing**

When a study contained more than one arm with the same studyID, agent, and dose, these duplicate arms were combined before constructing the MBNMAdose network. The combined effect estimate was calculated using

inverse-variance weighting, with weights defined as 1/SE^2. The combined SE was calculated as sqrt(1/sum(weights)), and sample sizes were summed.

This rule was applied to avoid treating identical study-agent-dose nodes as separate competing treatments within the same network model.

**3. Single-arm exclusions after preprocessing**

After duplicate-arm processing, studies that no longer contained at least two study arms were excluded from the

corresponding network analysis, because single-arm studies do not contribute comparative evidence to

NMA/MBNMAdose. In the CRP overall exercise dose-node analysis, studies 21 and 36 were excluded after preprocessing. In the IL-6 overall exercise dose-node analysis, study 29 was excluded after preprocessing.

**4. MBNMAdose model settings**

Common-effect and random-effects MBNMAdose models were [fitted using mbnma.network](fittedusingmbnma.network)() [and mbnma.run](andmbnma.run)()

from the MBNMAdose package. A second-degree polynomial dose-response function, dpoly(degree = 2), was used for the Hellinger distance comparison so that the supplementary Bayesian heterogeneity assessment aligned with the quadratic dose-response specification used for the primary MBNMAdose interpretation.

Each model used three MCMC chains, 20,000 iterations, 10,000 burn-in iterations, and a thinning interval of 10, yielding 3,000 retained posterior samples per model. MCMC performance was assessed using trace plots,

autocorrelation plots, Rhat statistics, and effective sample sizes.

**5. Hellinger distance calculation**

Hellinger distance (D) was calculated directly from posterior beta.1 and beta.2 samples from the common-effect and random-effects quadratic MBNMAdose models. D ranges from 0 to 1. Larger values indicate greater posterior

distributional divergence after allowing for between-study heterogeneity. Therefore, D was interpreted as a Bayesian distributional heterogeneity metric rather than as a transformation of frequentist I-squared.

**6. Software environment**

```
R version 4.6.0 (2026-04-24 ucrt)
Platform: x86_64-w64-mingw32/x64
Running under: Windows 11 x64 (build 26200)
Matrix products: default LAPACK version 3.12.1
locale:
[1] LC_COLLATE=Chinese (Simplified)_China.utf8
[2] LC_CTYPE=Chinese (Simplified)_China.utf8
[3] LC_MONETARY=Chinese (Simplified)_China.utf8
[4] LC_NUMERIC=C
[5] LC_TIME=Chinese (Simplified)_China.utf8 time zone: Asia/Seoul
tzcode source: internal
attached base packages:
[1] stats     graphics  grDevices utils     datasets  methods   base other attached packages:
[1] R2jags_0.8-9    rjags_4-17      coda_0.19-4.1   MBNMAdose_0.5.0 loaded via a namespace (and not attached):
[1] lattice_0.22-9   magrittr_2.0.5   R2WinBUGS_2.1-24 glue_1.8.1
[5] stringr_1.6.0    abind_1.4-8      parallel_4.6.0   lifecycle_1.0.5
[9] Rdpack_2.6.6     cli_3.6.6        grid_4.6.0       compiler_4.6.0
[13] boot_1.3-32      rbibutils_2.4.1  tools_4.6.0      rlang_1.2.0
[17] stringi_1.8.7
```

**Supplementary File S9. IL-6 Influence Analysis Excluding Ali Ismail 2025**

Purpose: This supplementary file documents the data audit and Bayesian sensitivity analysis requested by the reviewer for the potentially influential MBE study Ali Ismail 2025.

# S9.1 Data Verification

| **studyID** | **agent** | **dose** | **y** | **se** | **n** |
| --- | --- | --- | --- | --- | --- |
| 3 | 1 | 0 | 1.2 | 1.672 | 25 |
| 3 | 5 | 900 | -14.08 | 1.356 | 25 |

Interpretation: The MBE value of -14.08 was treated as a change-from-baseline value in the model-ready IL-6 dataset. It is consistent with the extracted values in the supplementary characteristics table (19.12 to 5.04), and no log transformation was applied.

# S9.2 Direct Contrast

| **studyID** | **treatment_agent** | **treatment_dose** | **treatment_y** | **treatment_se** | **control_y** | **control_se** | **contrast_y** | **contrast_se** | **contrast_z** |
| --- | --- | --- | --- | --- | --- | --- | --- | --- | --- |
| 3 | 5 | 900 | -14.08 | 1.356 | 1.2 | 1.672 | -15.28 | 2.152747 | -7.097908 |

The direct contrast was extremely large (z = -7.10), supporting reviewer concern that this study could drive the IL-6 network estimate and treatment ranking.

# S9.3 Ranking Sensitivity

| **analysis** | **treatment** | **median_effect** | **q2.5** | **q97.5** | **prob_best** | **mean_rank** | **SUCRA** |
| --- | --- | --- | --- | --- | --- | --- | --- |
| IL6_modality_full | 4_1500 | -0.992136 | -6.558011 | 4.456428 | 0.011 | 9.097889 | 0.523654 |
| IL6_modality_full | 5_300 | -1.254698 | -10.688799 | 8.028087 | 0.085778 | 8.891444 | 0.535797 |
| IL6_modality_full | 5_900 | -5.498949 | -12.025585 | 0.938517 | 0.291222 | 3.627444 | 0.845444 |
| IL6_modality_full | 5_1200 | -1.108667 | -9.989851 | 7.766254 | 0.063333 | 9.084444 | 0.524444 |
| IL6_modality_excluding_Ali_Ismail_2025 | 4_1500 | -1.014746 | -5.817217 | 3.885235 | 0.021889 | 8.333222 | 0.568634 |
| IL6_modality_excluding_Ali_Ismail_2025 | 5_300 | -1.23774 | -9.435171 | 6.859605 | 0.113556 | 8.303667 | 0.570373 |
| IL6_modality_excluding_Ali_Ismail_2025 | 5_900 | 0.619769 | -6.667478 | 7.738782 | 0.034667 | 10.945667 | 0.414961 |
| IL6_modality_excluding_Ali_Ismail_2025 | 5_1200 | -1.012024 | -8.7863 | 6.521297 | 0.092222 | 8.500889 | 0.558771 |

Key result: after excluding Ali Ismail 2025, the MBE 900 MET-min.week-1 node changed from SUCRA 0.845 to 0.415 and the probability of being best changed from 0.291 to 0.035. Therefore, the primary MBE ranking for IL-6 is not robust.

# S9.4 Key Nodes After Exclusion

| **analysis** | **treatment** | **median_effect** | **q2.5** | **q97.5** | **prob_best** | **SUCRA** |
| --- | --- | --- | --- | --- | --- | --- |
| IL6_modality_full | 3_900 | -1.553275 | -5.360501 | 2.270963 | 0.003556 | 0.588209 |
| IL6_modality_full | 5_300 | -1.254698 | -10.688799 | 8.028087 | 0.085778 | 0.535797 |
| IL6_modality_full | 5_900 | -5.498949 | -12.025585 | 0.938517 | 0.291222 | 0.845444 |
| IL6_modality_full | 5_1200 | -1.108667 | -9.989851 | 7.766254 | 0.063333 | 0.524444 |
| IL6_modality_full | 6_900 | -2.013816 | -11.833713 | 7.979478 | 0.125778 | 0.584458 |
| IL6_modality_excluding_Ali_Ismail_2025 | 3_900 | -1.449614 | -4.794769 | 1.864942 | 0.011222 | 0.627431 |
| IL6_modality_excluding_Ali_Ismail_2025 | 5_300 | -1.23774 | -9.435171 | 6.859605 | 0.113556 | 0.570373 |
| IL6_modality_excluding_Ali_Ismail_2025 | 5_900 | 0.619769 | -6.667478 | 7.738782 | 0.034667 | 0.414961 |
| IL6_modality_excluding_Ali_Ismail_2025 | 5_1200 | -1.012024 | -8.7863 | 6.521297 | 0.092222 | 0.558771 |
| IL6_modality_excluding_Ali_Ismail_2025 | 6_900 | -2.047892 | -10.726546 | 6.797095 | 0.169111 | 0.630895 |

# S9.5 MBNMAdose Parameter Sensitivity

| **analysis** | **parameter** | **median** | **q2.5** | **q97.5** | **prob_less_than_0** | **prob_greater_than_0** |
| --- | --- | --- | --- | --- | --- | --- |
| Full data | beta.1[5] | -0.014121 | -0.03911 | 0.010839 | 0.876889 | 0.123111 |
| Full data | beta.2[5] | 1e-05 | -1.3e-05 | 3.4e-05 | 0.180889 | 0.819111 |
| Excluding Ali Ismail 2025 | beta.1[5] | 0.000595 | -0.022361 | 0.024035 | 0.478667 | 0.521333 |
| Excluding Ali Ismail 2025 | beta.2[5] | -1e-06 | -2.3e-05 | 2.1e-05 | 0.529778 | 0.470222 |

# S9.6 Model Diagnostics

| **analysis** | **DIC** | **Rhat_max** | **n_eff_min** | **n_studies** | **n_arms** | **sd_median** | **sd_q2.5** | **sd_q97.5** |
| --- | --- | --- | --- | --- | --- | --- | --- | --- |
| IL6_modality_full_MBNMAdose | 134.601191 | 1.001237 | 1 | 32 | 71 | 4.093076 | 3.084067 | 5.595907 |
| IL6_modality_excluding_Ali_Ismail_2025_MBNMAdose | 121.597087 | 1.003536 | 1 | 31 | 69 | 3.557504 | 2.717834 | 4.873836 |

# S9.7 MCMC Trace and Autocorrelation Plots

Supplementary Figure S9-1. Full-data IL-6 modality-level quadratic MBNMAdose trace and autocorrelation diagnostics.

Supplementary Figure S9-2. IL-6 modality-level quadratic MBNMAdose trace and autocorrelation diagnostics after excluding Ali Ismail 2025.
